# Scalable microfluidic synthesis of noble metal single-atom catalysts

**DOI:** 10.1093/nsr/nwag276

**Published:** 2026-05-15

**Authors:** Jingyi Tian, Cao Zhou, Biao Feng, Xiaoyu Liu, Mengyu Deng, Hui Yang, Hongwen Huang, Xizhang Wang, Qiang Wu, Lijun Yang, Zheng Hu

**Affiliations:** State Key Laboratory of Coordination Chemistry and Key Laboratory of Mesoscopic Chemistry of MOE, School of Chemistry and Chemical Engineering, Nanjing University, Nanjing 210023, China; State Key Laboratory of Coordination Chemistry and Key Laboratory of Mesoscopic Chemistry of MOE, School of Chemistry and Chemical Engineering, Nanjing University, Nanjing 210023, China; State Key Laboratory of Coordination Chemistry and Key Laboratory of Mesoscopic Chemistry of MOE, School of Chemistry and Chemical Engineering, Nanjing University, Nanjing 210023, China; State Key Laboratory of Coordination Chemistry and Key Laboratory of Mesoscopic Chemistry of MOE, School of Chemistry and Chemical Engineering, Nanjing University, Nanjing 210023, China; State Key Laboratory of Coordination Chemistry and Key Laboratory of Mesoscopic Chemistry of MOE, School of Chemistry and Chemical Engineering, Nanjing University, Nanjing 210023, China; Shanghai Advanced Research Institute, Chinese Academy of Sciences, Shanghai 201210, China; State Key Laboratory of Coordination Chemistry and Key Laboratory of Mesoscopic Chemistry of MOE, School of Chemistry and Chemical Engineering, Nanjing University, Nanjing 210023, China; State Key Laboratory of Coordination Chemistry and Key Laboratory of Mesoscopic Chemistry of MOE, School of Chemistry and Chemical Engineering, Nanjing University, Nanjing 210023, China; State Key Laboratory of Coordination Chemistry and Key Laboratory of Mesoscopic Chemistry of MOE, School of Chemistry and Chemical Engineering, Nanjing University, Nanjing 210023, China; State Key Laboratory of Coordination Chemistry and Key Laboratory of Mesoscopic Chemistry of MOE, School of Chemistry and Chemical Engineering, Nanjing University, Nanjing 210023, China; State Key Laboratory of Coordination Chemistry and Key Laboratory of Mesoscopic Chemistry of MOE, School of Chemistry and Chemical Engineering, Nanjing University, Nanjing 210023, China

**Keywords:** microfluidic synthesis, noble metal single-atom catalysts, mass production, nitrogen-doped carbon nanocages, proton exchange membrane water electrolysis

## Abstract

Scalable synthesis of noble metal single-atom catalysts (SACs) is crucial for practical applications but remains challenging due to the ‘scaling-up effect’ by spatiotemporal variations of concentration/temperature, leading to poor uniformity and/or metal aggregation. Here, we report a continuous-flow microfluidic strategy for mass production of Pt SACs on hierarchical nitrogen-doped carbon nanocages (Pt_1_/hNCNC), which fundamentally overcomes this limitation. Combined experiments and theoretical simulations indicate that the microfluidic microreactor efficiently avoids local supersaturation via rapid and uniform mixing. In synergy with micropore trapping and nitrogen anchoring of hNCNC, continuous local adsorption equilibrium is maintained, promoting stable immobilization of isolated atoms while suppressing aggregation. This method achieves scalable synthesis of Pt_1_/hNCNC SACs with high Pt loading (10 wt%), excellent uniformity, and a nearly 300-fold increase in productivity compared to the batch method. When applied in a proton exchange membrane water electrolyzer, the cathode with an ultralow loading of 20 μg_Pt_ cm^−2^ delivers industrial current densities of 1.0/3.0 A cm^−2^ at low voltages of 1.66/2.00 V, respectively, and operates stably for over 500 h at 1.0 A cm^−2^. This microfluidic synthesis is also successfully extended to various noble metals (Pd, Au, Ir, Rh, Ru, and multi-elements) and nitrogen-doped carbons, demonstrating broad generality. This work establishes a practical and scalable paradigm for manufacturing high-performance noble metal SACs with deep insights into the dual-scale cooperation between macroscopic fluid dynamics and microscopic support chemistry, bridging laboratory discovery with industrial application of single-atom catalysis.

## Teaser

A continuous-flow microfluidic synthesis is developed to overcome the scaling-up effect, enabling mass production of noble metal single-atom catalysts on hierarchical nitrogen-doped carbon nanocages with exceptional uniformity and industrial potential.

## INTRODUCTION

The advent of single-atom catalysts (SACs) has ushered in a new era in heterogeneous catalysis, offering the ultimate in atom utilization efficiency and unlocking catalytic properties that are often unattainable with their nanoparticle (NP) counterparts [1–3]. This transformation is particularly crucial for noble metals, where scarcity and cost are perennial concerns, as SACs promise to maximize the performance of every single atom. Despite tremendous progress in synthetic methodologies, including atomic layer deposition [4,5], high-temperature pyrolysis or thermal migration [6,7], and a host of other elegant techniques [8–13], the field is currently at a critical turning point. The core challenge has shifted from lab-scale material fabrication to industrial translation, i.e. how to simultaneously realize the scalable, structurally precise, and batch-stable/reproducible synthesis of noble metal SACs [14,15]. Traditional batch-type syntheses, while effective on a milligram scale, are plagued by a formidable ‘scaling-up effect’. As the reactor volume expands, the inherent limitations of mass and heat transfer lead to spatiotemporal inhomogeneities in precursor concentration and temperature. These nonuniform local environments form supersaturation ‘hotspots’, which uncontrollably drive the migration and agglomeration of isolated metal atoms into catalytically distinct nanoclusters (NCs)/NPs. This loss of single-atom fidelity not only degrades performance but also leads to irreproducible results, erecting a seemingly insurmountable barrier between promising laboratory findings and practical applications. Thus, the critical question nowadays is how to fundamentally overcome the physical laws of mass/heat transport that govern aggregation during scaling-up to achieve a general, scalable synthesis of uniform, high-performance noble metal SACs [2,16].

As we know, microfluidic synthesis represents an advanced methodology owing to its precise manipulation of fluids within micron- to millimeter-scale channels, distinguished by superior capabilities in reaction environment control, mass transfer efficiency, reagent utilization, and scalability [17]. Over the past two decades, microfluidic synthesis has been widely used to produce NP catalysts with controlled morphology and size [18,19]. Unfortunately, the very limited experimental explorations on the microfluidic synthesis of SACs have not achieved gram-scale production, largely due to complex setups, high energy consumption, or ultralow microliter-scale throughput [20–22]. Therefore, the continuous-flow synthesis strategy which features simple equipment, a well-defined mechanism, and genuine scalability remains an urgent need in SACs field. Notably, the excellent mass and heat transfer in microfluidic reactors only provides external conditions for scalable SAC production. To fundamentally suppress metal aggregation and realize stable high‑loading anchoring, the intrinsic properties of the support are equally essential for strong precursor capture and robust anchoring. In recent years, with unique hierarchical nitrogen-doped carbon nanocages (hNCNC) [23,24], we developed the simplest impregnation-adsorption method for noble metal SAC construction via the synergism of micropore trapping and nitrogen anchoring [23,24], yielding Pt_1_/hNCNC with a maximum Pt_1_ loading of ca. 5.68 wt% and excellent hydrogen evolution reaction (HER) performance [24,25]. In this work, we present a transformative solution to scalable microfluidic synthesis of noble metal SACs by combining the merits of the impregnation-adsorption method, using the synthesis of Pt_1_/hNCNC as our primary model. Our central hypothesis is that the unique mass-transfer environment of a microfluidic reactor can establish a continuous local adsorption equilibrium, thereby circumventing the transient supersaturation that triggers aggregation in batch processes. By integrating multiscale theoretical simulations with comprehensive experimental validation, we elucidate a ‘dual-scale cooperation’ between macroscopic fluid dynamics and microscopic support chemistry that enables this paradigm shift. This approach achieves a remarkable 300-fold increase in productivity over the batch counterpart, pushes the maximum Pt_1_ loading up to 10 wt%, and demonstrates exceptional generality across a suite of noble metals (Pd, Au, Ir, Rh, Ru, and multi-elements) and nitrogen-doped carbon supports. Crucially, ultralow loading (20 μg_Pt_ cm^−2^) of flow-synthesized 10 wt%-Pt_1_/hNCNC on cathode ensures stable operation of proton exchange membrane water electrolyzers (PEMWEs) over 500 h at industrial current densities, validating the industrial potential of our scalable synthesis. This study establishes a scalable and versatile paradigm for manufacturing noble metal SACs, effectively bridging the critical gap between laboratory discovery and industrial application in the field of single-atom catalysis.

## RESULTS AND DISCUSSION

### Microfluidic synthesis of Pt_1_/hNCNC

#### Synthesis setup and scalability

**Figure 1.**
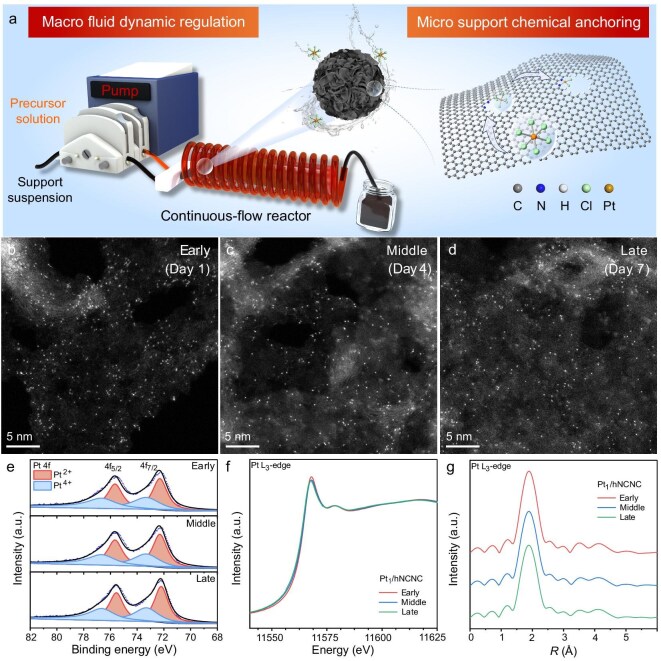
Structural uniformity of Pt_1_/hNCNC via microfluidic synthesis. (a) Schematic illustration of microfluidic synthesis. (b–d) HAADF-STEM images of Pt_1_/hNCNC collected at early, middle, and late synthetic stages. (e) Pt 4f spectra. (f) Normalized XANES spectra at the Pt L_3_ edge. (g) *k*^2^-weighted R-space FT-EXAFS.

Figure 1 illustrates the schematic diagram of microfluidic synthesis and structural uniformity of the obtained Pt_1_/hNCNC. The hNCNC support has abundant and highly accessible anchoring sites, favorable to the high dispersion of Pt atoms (Fig. S1) [24,26]. The home-made microfluidic reactor is composed of a peristaltic pump and a polytetra-fluoroethylene (PTFE) tube (inner diameter: 1 mm; length: 10 m) with a temperature controller (Fig. 1a and Fig. S2). Briefly, the H_2_PtCl_6_ aqueous solution and hNCNC suspension are separately pumped at the predetermined flow rate (e.g. 15 mL min^−1^) and then uniformly mixed in the PTFE tube. The mixture subsequently passes through a temperature-controlled zone (e.g. 60°C) for a specified retention time (e.g. 3 min) to achieve the uniform anchoring of Pt SACs, followed by filtrating, washing, and drying, leading to the formation of Pt_1_/hNCNC (see detailed synthetic conditions in Table S1). By using the hNCNC support and metal precursor scaled up proportionally to the quantities utilized in the flask-scale impregnation-adsorption process [24], we successfully prepared 200 g of Pt_1_/hNCNC with a nominal Pt loading of 3 wt% within 1 week, which translates to a nearly 300-fold increase in productivity (Figs S2 and S3). Compared with earlier systems (complex, high energy consumption, low throughput) [20–22], our setup is simple, mild (60°C), and enables high throughput (30 g per day), realizing genuine scalability.

#### Structural uniformity and maximum loading

To assess product uniformity across the continuous-flow process, samples were collected at the early (day 1), middle (day 4), and late (day 7) stages for comprehensive characterization. Inductively coupled plasma-optical emission spectrometry (ICP-OES) reveals highly consistent Pt contents of 2.96, 3.02, and 3.01 wt%, respectively. High-angle annular dark-field scanning transmission electron microscopy (HAADF-STEM) images confirm atomic dispersion of Pt species in all samples, without observable Pt NCs/NPs, consistent with the absence of Pt characteristic peaks in corresponding X-ray diffraction (XRD) patterns (Fig. 1b–d and Fig. S4). The Pt 4f fine spectra of X-ray photoelectron spectroscopy (XPS) exhibit identical features, indicating consistent chemical composition (Fig. 1e). X-ray absorption near edge structure (XANES) spectroscopy at the Pt L_3_ edge shows matching absorption edge positions and white line (WL) intensities, confirming uniform Pt valence states across the entire synthesis process (Fig. 1f). Fourier-transform extended X-ray absorption fine structure (FT-EXAFS) spectra consistently lack Pt–Pt coordination signals at 2.61 Å, further verifying the same single-atom structure of Pt_1_/hNCNC (Fig. 1g). These self-consistent characterization results demonstrate that the Pt_1_/hNCNC catalysts obtained at different stages have high uniformity in Pt loading, dispersion state, chemical valence, and coordination structure. This uniformity ensures consistent catalytic performance regardless of preparation scale (Fig. S4), which is a critical advantage for practical applications. Collectively, these findings confirm that our continuous-flow microfluidic method achieves the mass synthesis of highly uniform Pt_1_/hNCNC catalysts and effectively overcomes the limitations of conventional flask-based impregnation-adsorption method [24,25].

**Figure 2.**
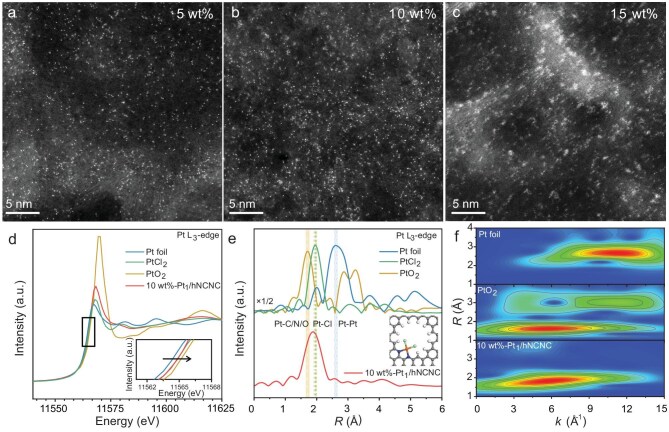
Maximum loading of Pt_1_/hNCNC via microfluidic synthesis. HAADF-STEM images of 5 wt%-Pt_1_/hNCNC (a), 10 wt%-Pt_1_/hNCNC (b), and 15 wt%-Pt/hNCNC (c). Normalized Pt L_3_-edge XANES spectra (d) and *k*^2^-weighted R-space FT-EXAFS (e) for 10 wt%-Pt_1_/hNCNC, with Pt foil, PtCl_2_, and PtO_2_ for reference. (f) WT-EXAFS of 10 wt%-Pt_1_/hNCNC, Pt foil, and PtO_2_.

We next explored the maximum achievable single-atom loading by increasing the H_2_PtCl_6_ precursor concentration, producing catalysts with nominal Pt loadings of 5, 10, and 15 wt%. HAADF-STEM images of the 5 and 10 wt% samples show uniformly distributed isolated bright spots on hNCNC, with spot density increasing alongside Pt content (Fig. 2a and b). At 15 wt%, however, a small number of subnanometer NCs (<1 nm) appear alongside the dominant single-atom sites (Fig. 2c and Fig. S5). For the 10 wt% sample, the Pt loading was quantitatively confirmed as 9.66 wt% by ultraviolet-visible absorption spectroscopy, 10.14 wt% by thermogravimetric analysis, and 9.73 wt% by ICP-OES, all consistent with the nominal 10 wt% target (Fig. S6). This result indicates that the Pt single-atom loading threshold for Pt_1_/hNCNC synthesized by the microfluidic method can reach 10 wt%, significantly higher than the 5.68 wt% limit of the batch impregnation-adsorption process (Fig. S7) [24].

A detailed analysis of the 10 wt%-Pt_1_/hNCNC was performed to elucidate its chemical state and coordination structure. The XANES WL intensity positions between Pt foil and PtO_2_ references, verifying the oxidation state of Pt species with average valence of ca. +1.83 (Fig. 2d and Fig. S8) [27]. The FT-EXAFS spectrum features a main peak at ∼1.87 Å attributed to combined Pt-C/N and Pt-Cl coordination shells, without evident Pt–Pt bonds at 2.61 Å (Fig. 2e and Fig. S9) [28,29]. Wavelet transform (WT) analysis displays only one intensity maximum at ∼5.7 Å⁻^1^ (backscattering from light atoms) and none at ∼11.2 Å⁻^1^ (characteristic of Pt–Pt coordination), corroborating the exclusive single-atom dispersion (Fig. 2f) [30]. Combining EXAFS fitting and density functional theory (DFT) simulation, the local coordination of Pt single atoms is determined to be Pt-N_2_Cl_2_ (Table S2) [25]. These results are also supported by some other routine characterizations (Figs S10 and S11).

**Figure 3.**
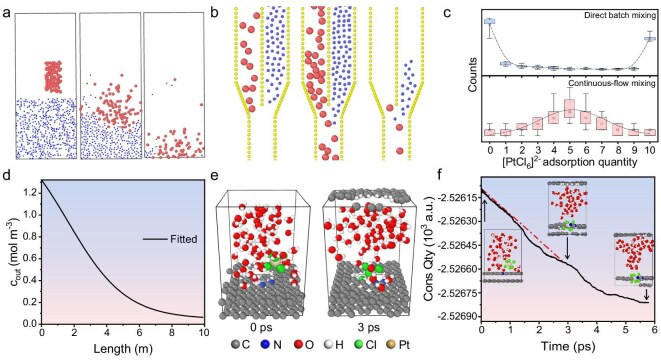
Theoretical modeling of batch and microfluidic synthesis. The initial, proceeding, and ending frames of MD simulation of direct batch mixing (a) and Y-shaped microchannel microfluidic mixing (b). (c) Statistical histograms of the adsorption quantity of [PtCl_6_]^2^⁻ on each hNCNC, including 12 independent MD simulations. The box limits indicate the upper and lower quartiles, the whiskers extend to 1.5× the interquartile range, the central small box represents the median. (d) The [PtCl_6_]^2^⁻ concentration along the continuous-flow reactor. AIMD simulation on the capture of a [PtCl_6_]^2−^ anion by graphitic bilayer with a micropore decorated by two pyridinic-N atoms: perspective-view configurations at 0 and 3 ps (e) and total energy evolution (f). In panel (f), dashed line represents the expected trend when [PtCl_6_]^2−^ has not yet entered the micropore. Insets correspond to front-view configurations of the system at the respective time points.

The preceding results demonstrate that the microfluidic synthesis successfully overcomes traditional scalability limits, enabling mass production of uniform Pt_1_/hNCNC with single-atom loading up to 10 wt%. To understand the origin of this improved uniformity and loading, we performed molecular dynamics (MD) simulations comparing different mixing modes (Fig. 3). In simulations, the mixing-adsorption process is simplified into a coarse-grained model that captures the essential features of precursor transport and local adsorption. The model system comprises 100 hNCNC particles (red spheres) and 500 [PtCl_6_]^2^⁻ ions (blue particles). At fixed time intervals (every 500 MD steps), each hNCNC irreversibly adsorbs all [PtCl_6_]^2^⁻ ions appeared within its first-neighbor coordination shell, mimicking diffusion-limited capture. Adsorbed ions per hNCNC were recorded, with a 10-ion maximum to represent saturation of anchoring sites (see the Experimental section). Figure 3a illustrates the mixing mode characteristic of the traditional batch impregnation-adsorption process, in which hNCNC spheres are immersed into a stationary [PtCl_6_]^2^⁻ pool (Video S1). This adsorption process exhibits a bimodal peak at the adsorbed number of 0 (empty) and 10 (saturated), resulting in a poor uniformity of Pt atom dispersion on hNCNC (Fig. 3c). In contrast, the continuous-flow mixing in a Y-shaped microchannel demonstrates superior uniformity. Here, each hNCNC sphere only meets a specific number of precursor ions each time (Fig. 3b and Video S2). Statistical analysis reveals a narrow Gaussian distribution centered at the ideal loading of five ions per support, effectively ensuring uniform adsorption and Pt atom dispersion (Fig. 3c). These comparative results demonstrate that the microfluidic method efficiently enhances mixing homogeneity, avoids local supersaturation, and thereby inhibits the Pt aggregation into NCs/NPs, as reflected by the increased Pt single-atom loading threshold to 10 wt% (from 5.68 wt% for batch process) (Fig. 2b and Fig. S7). Finite element method calculation was conducted to simulate the mass transfer and adsorption behaviors of [PtCl_6_]^2^⁻ ions within the microfluidic reactor. The fitted [PtCl_6_]^2^⁻ concentration profile exhibits a progressive decay along the reactor length, avoiding the drastic concentration change or local supersaturation in traditional batch reaction (Fig. 3d, Fig. S12, and Table S3). Hence, in any microsegment, the diffusion rate of [PtCl_6_]^2^⁻ toward the hNCNC surface and the adsorption rate on the hNCNC surface achieve instantaneous equilibrium. Such continuous local adsorption equilibrium states suppress Pt species aggregation and guarantee high product uniformity.

At the molecular level, the rapid adsorption of [PtCl_6_]^2^⁻ originate from the synergistic effect of micropore trapping and N-atom anchoring in hNCNC support (Figs S13 and S14 and Table S4) [24]. Ab initio molecular dynamics (AIMD) simulations were performed to examine the capture of a freely diffusing [PtCl_6_]^2−^ anion by a functionalized micropore on hNCNC with Model 5 (Fig. S13). The [PtCl_6_]^2^⁻ anion, initially placed outside the micropore of the model, is rapidly drawn into the pore within 3 ps due to electrostatic attraction from the protonated pyridinic-N sites, which shortens the minimal distance between Pt and N from 6.72 to 4.97 Å (Fig. 3e). This process was accompanied by a pronounced decrease in total energy, indicating spontaneous and energetically favorable trapping (Fig. 3f). Once trapped in the pore, the system quickly approaches a stable equilibrium state (Fig. S15 and Video S3). These results provide direct atomic-scale evidence that functionalized micropores enable both efficient precursor capture and stable anchoring.

Overall, the continuous-flow synthesis enables scalable, uniform production of Pt_1_/hNCNC via the synergy of macroscopic microfluidic reactor control and microscopic adsorption enhancement. At the macro scale, the microfluidic reactor ensures homogeneous precursor mixing and continuous local adsorption equilibrium, effectively suppressing uneven loading and Pt aggregation (Fig. 3a–d and Fig. S12). At the molecular level, hNCNC’s hierarchical structure (abundant micropores and pyridinic‑N dopants) provides kinetic confinement and thermodynamic stabilization for [PtCl_6_]^2−^ (Fig. 3e and f and Figs S13–S15). This dual-scale cooperation shortens precursor free diffusion time, promotes rapid and uniform anchoring, and ultimately achieves Pt single-atom loading up to 10 wt% with high consistent quality across batches. This mechanistic insight offers a generalizable design principle for scalable SAC synthesis.

### Generalized application of the microfluidic synthesis

**Figure 4.**
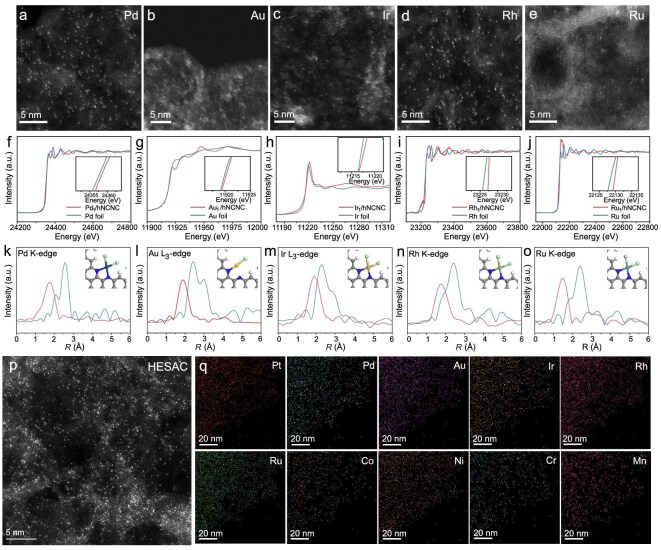
Morphology and structure characterizations of M_1_/hNCNC (M = Pd, Au, Ir, Rh, and Ru) and HESAC. (a–e) HAADF-STEM images of M_1_/hNCNC. (f–j) Normalized XANES spectra. (k–o) *k*^2^-weighted R-space FT-EXAFS. Insets are the corresponding optimized structures. HAADF-STEM (p) and EDS elemental mapping images (q) of HESAC.

The versatility of our microfluidic platform was demonstrated by synthesizing a series of noble metal SACs of M_1_/hNCNC (M = Pd, Au, Ir, Rh, and Ru) using PdCl_2_, HAuCl_4_, H_2_IrCl_6_, RhCl_3_, and RuCl_3_ as respective metal precursors (Table S1). HAADF-STEM images display uniformly dispersed bright spots smaller than 0.2 nm, confirming the atomic dispersion of each metal (Fig. 4a–e). XANES spectra verify the oxidation states of metal species in M_1_/hNCNC as reflected by the higher absorption edge positions relative to corresponding M foils (Fig. 4f–j). All the FT-EXAFS spectra exhibit a main peak around 1.5∼1.9 Å, without obvious M–M scattering near 2.6 Å, confirming the single-atom nature of M_1_/hNCNC. Combined EXAFS fitting and DFT calculations suggest the local coordination structures of Pd-N_2_Cl_2_, Au-N_1_Cl_1_, Ir-N_2_Cl_2_, Rh-N_2_Cl_2_, and Ru-N_2_Cl_2_, respectively (Fig. 4k–o). XRD, XPS, transmission electron microscopy (TEM), and energy dispersive spectrometer (EDS) mapping characterizations further confirm the uniform spatial distribution of positively charged isolated metal sites on hNCNC, with no detectable metal aggregation (Figs S16 and S17). ICP-OES quantifies the metal loadings as 2.70 wt% (Pd), 1.38 wt% (Au), 2.92 wt% (Ir), 2.81 wt% (Rh), and 1.53 wt% (Ru). The near-theoretical loadings (∼3.0 wt%) achieved for Pd, Ir, and Rh are attributed to the strong affinity of their complex anions for the N-containing micropores of hNCNC (Fig. S13a and Table S4). The lower loadings of Au and Ru could be associated with the precursor hydrolysis or reduction, which decreases their effective concentration during the microfluidic process.

To further showcase the method’s capability, we synthesized a high-entropy SAC (HESAC) containing six noble metals (Pt, Pd, Au, Ir, Rh, and Ru) on a prefunctionalized hNCNC support. HAADF-STEM image reveals a high density of atomically dispersed species without visible clusters (Fig. 4p). EDS elemental mapping confirms the homogeneous distribution of all metals throughout the support (Fig. 4q). All metal species in HESAC are in oxidation states (Fig. S18).

**Figure 5.**
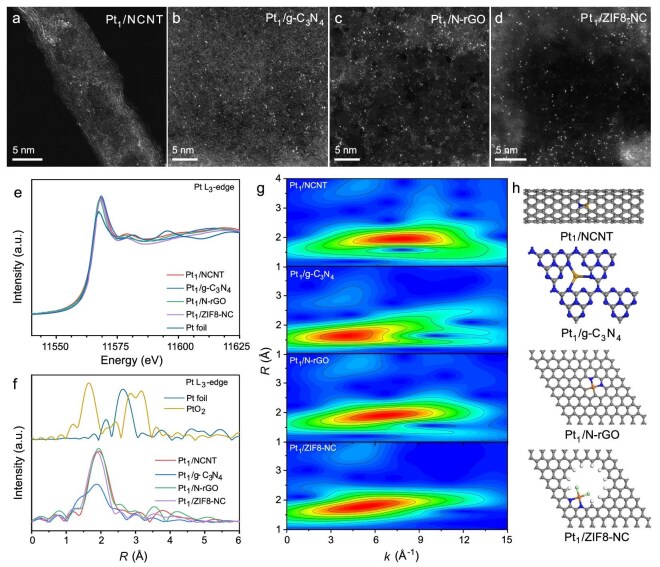
Morphology and structure characterizations of Pt_1_/NC (NC = NCNT, g-C_3_N_4_, N-rGO, and ZIF8-NC). (a–d) HAADF-STEM images. (e) Normalized XANES spectra at the Pt L_3_ edge. (f) *k*^2^-weighted R-space FT-EXAFS. (g) WT-EXAFS. (h) Optimized structures.

The microfluidic synthesis was also successfully applied to anchor Pt single atoms on various other nitrogen-doped carbon supports, including N-doped carbon nanotubes (NCNT), g-C_3_N_4_, N-doped reduced graphene oxide (N-rGO), and ZIF8-derived N-doped carbon (ZIF8-NC). Similarly, HAADF-STEM images show bright spots smaller than 0.2 nm, confirming successful Pt single-atom formation on all supports (Fig. 5a–d and Fig. S19a–c). XANES spectra exhibit higher WL intensities than Pt foil, confirming an oxidized Pt state (Fig. 5e and Fig. S19d and e). FT-EXAFS spectra feature a main peak near 1.85 Å (Pt-C/N coordination) without obvious Pt–Pt contribution at 2.61 Å, and WT analysis further evidenced the exclusive Pt–light atom coordination (Fig. 5f and g). EXAFS fitting and DFT calculations elucidate the local coordination structures of Pt-C*_x_*N_3−_*_x_* (0 ≤ *x* ≤ 3) for Pt_1_/NCNT, Pt-N_3_ for Pt_1_/g-C_3_N_4_, Pt-C*_x_*N_4−_*_x_* (0 ≤ *x* ≤ 4) for Pt_1_/N-rGO, and Pt-N_2_Cl_2_ for Pt_1_/ZIF8-NC (Fig. 5h and Fig. S20). Although the theoretical Pt loading was identical (3.0 wt%), the actual Pt loadings measured by ICP-OES differed considerably owing to the distinct properties of the supports (Fig. S21). These results underscore the general applicability of the microfluidic method for preparing Pt SACs on various N-doped carbons, while support properties critically influence the final single-atom loading and coordination geometry. Hierarchical porous N-doped carbons with high N content and specific surface area prove most effective for anchoring high-loading noble metal single atoms.

The aforementioned results indicate that the microfluidic method enables large-scale synthesis of uniform noble metal SACs across a wide range of metals and supports, featured by simple operation and broad generality. With a daily production rate of 30 g per setup, i.e. 300-fold increase in productivity relative to batch synthesis, this approach establishes a scalable paradigm for the industrial preparation of high-performance noble metal SACs through parallelization (Fig. S2 and Note S1).

### Electrocatalytic HER performance

**Figure 6.**
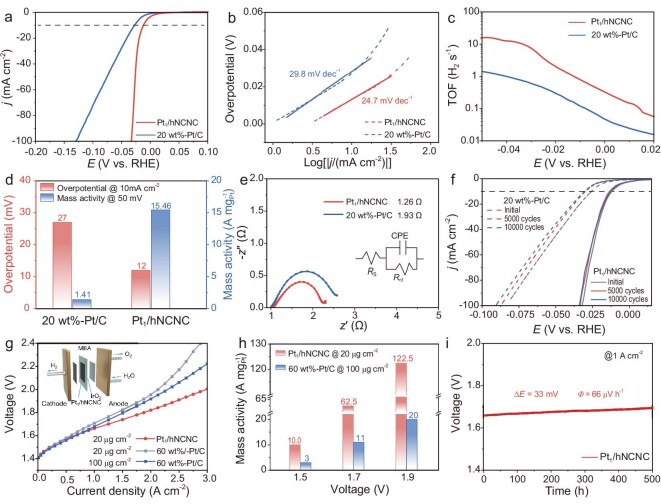
Electrocatalytic HER and PEMWE performances of 10 wt%-Pt_1_/hNCNC. HER performance in a three-electrode system: (a) LSV curves; (b) Tafel slopes [dotted lines are derived experimental data from panel (a); solid lines are fitted curves]; (c) TOF at different potentials; (d) *η*_10_ and mass activities at 50 mV; (e) Nyquist plots at 50 mV; (f) LSV curves before and after 5000 and 10 000 CV cycles. PEMWE performance: (g) polarization curves (inset is the schematic diagram of the PEMWE device); (h) Pt mass activity; (i) chronopotentiometry curve at 1.0 A cm^−2^.

The scalability and versatility of the microfluidic method lay a solid foundation for the practical applications of noble metal SACs. To demonstrate this, we evaluated the HER performance of the continuously produced 10 wt%-Pt_1_/hNCNC, targeting a drastic reduction in Pt usage for PEMWE from the commercial usage of 0.5–1.0 mg_Pt_ cm⁻^2^ [31,32]. First, HER activity was assessed in a three-electrode system with 0.5 M H_2_SO_4_ solution. Linear sweep voltammetry (LSV) curves show that the ultralow overpotentials (*η*) of only 12 and 33 mV are required to achieve current densities of 10 (*η*_10_) and 100 mA cm^−2^ (*η*_100_), respectively, outperforming commercial 20 wt% Pt/C (*η*_10_ = 27 mV, *η*_100_ =130 mV) (Fig. 6a). The Pt_1_/hNCNC exhibits a lower Tafel slope (24.7 mV dec^−1^) than Pt/C (29.8 mV dec^−1^), implying a Volmer–Tafel HER mechanism (Fig. 6b) [33,34]. At 50 mV, the intrinsic activity, reflected by the turnover frequency (TOF), reaches 15.46 H_2_ s^−1^, far exceeding corresponding 1.41 H_2_ s^−1^ of Pt/C (Fig. 6c). This results in ∼11 times higher mass activity of Pt_1_/hNCNC than Pt/C, highlighting exceptional Pt utilization efficiency (Fig. 6d). The favorable HER kinetics of Pt_1_/hNCNC is further corroborated by electrochemical impedance spectroscopy, showing a lower charge transfer resistance (*R*_ct_ = 1.26 Ω) than that of Pt/C (*R*_ct_ = 1.93 Ω) (Fig. 6e) [35]. As established in our recent studies, the high activity originates from the low-coordinated Pt-N_2_ active sites *in situ* formed from the dechlorination of Pt-N_2_Cl_2_ sites under HER working condition, which enable the multi-H adsorption mode with an ideal Δ*G*_H*_ of nearly 0 eV [25]. Accelerated durability test (ADT) shows excellent stability for Pt_1_/hNCNC, with a minimal increase of only 2.5 mV in *η*_100_ after 10 000 cyclic voltammetry (CV) scans, much better than commercial Pt/C (Δ*η*_100_ = 7 mV after 10 000 cycles) (Fig. 6f). After the ADT, Pt_1_/hNCNC maintains Pt single atoms without observable morphological change, confirming robust structural stability (Fig. S22) [36].

To assess practical viability, membrane–electrode–assembly (MEA) was fabricated using 10 wt%-Pt_1_/hNCNC or commercial 60 wt% Pt/C as the cathode for PEMWE. With an ultralow Pt loading of 20 μg_Pt_ cm^−2^, the Pt_1_/hNCNC-based MEA achieves current densities of 1.0 and 3.0 A cm^−2^ at low cell voltages of 1.66 and 2.00 V, respectively. This performance is superior to that of Pt/C-based MEA (1.0 A cm^−2^@1.68 V; 3.0 A cm^−2^@2.22 V), even though the latter used a five-times higher Pt loading (100 μg_Pt_ cm^−2^) (Fig. 6g). The mass activity of the Pt_1_/hNCNC MEA at 1.7 V reaches 62.5 A mg_Pt_^−1^, approximately six times higher than that of the Pt/C benchmark (11.0 A mg_Pt_^−1^) (Fig. 6h). This places our catalyst among the most competitive Pt-based SACs reported to date (Table S5). The excellent performance is attributed to the highly active Pt-N_2_ sites and the hierarchical, conductive hNCNC support that enables rapid mass/charge transfer [25]. Moreover, the Pt_1_/hNCNC MEA exhibits remarkable operational stability, sustaining 1.0 A cm^−2^ for over 500 h with a slight voltage increase of ∼33 mV (Fig. 6i and Fig. S23). Based on these results, the Pt_1_/hNCNC catalyst synthesized by the scalable microfluidic method exhibits exceptional HER activity, stability, and Pt utilization efficiency, even superior to the performance of catalysts made by lab-scale batch methods [25]. These results validate the microfluidic approach as a powerful and scalable strategy for producing high-performance SACs ready for industrial application.

## CONCLUSIONS

In summary, this study pioneers a continuous-flow microfluidic strategy to address the critical ‘scaling-up effect’ in noble metal SAC synthesis. Methodologically, this strategy demonstrates a ∼200 g production of uniform Pt_1_/hNCNC with a nearly 300-fold productivity increase over batch methods, reaching a high Pt loading up to 10 wt% (significantly higher than the 5.68 wt% limit of batch process). Mechanistically, integrated experiments and multiscale simulations reveal a dual-scale cooperation. Macroscopic microfluidic mixing prevents local supersaturation via continuous adsorption equilibrium, effectively suppressing uneven loading and Pt aggregation, while microscopic micropore trapping and nitrogen anchoring of hNCNC provide kinetic confinement and thermodynamic stabilization for [PtCl_6_]^2−^ anions. Practically, the utility of this scalable synthesis was decisively validated in a demanding industrial application. A PEMWE employing an ultralow cathode loading of 20 μgPt cm^−2^ of the continuously produced 10 wt%-Pt_1_/hNCNC delivers industrial-grade current densities of 1.0/3.0 A cm^−2^ at low cell voltages of 1.66/2.00 V, respectively, and exhibits exceptional long-term stability for over 500 h at 1.0 A cm^−2^. The strategy is readily extended to various noble metals (Pd, Au, Ir, Rh, Ru, and multi-elements) and nitrogen-doped carbons, demonstrating broad generality. Looking forward, integration of this microfluidic system with artificial intelligence and automated manufacturing will realize intelligent optimization, real-time regulation, and scalable industrial production of high-performance SACs. In a word, this work establishes a practical, scalable, and broadly applicable microfluidic paradigm for manufacturing high-quality noble metal SACs. It not only solves the long-standing scaling-up challenge but also offers deep mechanistic insights into the dual-scale cooperation between macroscopic fluid dynamics and microscopic support chemistry, thereby bridging laboratory discovery with industrial application of single-atom catalysis.

## Supplementary Material

nwag276_Supplemental_Files
