# Supplementary material for: Scalable microfluidic synthesis of noble metal single-atom catalysts
Source: Natl Sci Rev. 2026 May 15;13(15):nwag276. doi: 10.1093/nsr/nwag276 (PMC13455616; doi:10.1093/nsr/nwag276)
Supplement: Supplementary file 1 [file nwag276_supplemental_files.zip › SI-nsr-557.R1_.pdf]

Supporting Information

Scalable Microfluidic Synthesis of Noble Metal Single-Atom Catalysts

Jingyi Tian[1]ǂ, Cao Zhou[1]ǂ, Biao Feng[1], Xiaoyu Liu[1], Mengyu Deng[1], Hui Yang[2], Hongwen Huang[1], Xizhang Wang[1], Qiang Wu[1], Lijun Yang[1]*, Zheng Hu[1]**

1 State Key Laboratory of Coordination Chemistry and Key Laboratory of Mesoscopic Chemistry of MOE, School of Chemistry and Chemical Engineering, Nanjing University, Nanjing 210023, China.
2 Shanghai Advanced Research Institute, Chinese Academy of Sciences, 201210 Shanghai, China

*** Corresponding authors.**
Email: wqchem@nju.edu.cn; lijunyang@nju.edu.cn; zhenghu@nju.edu.cn

Methods

Chemicals

$H_2PtCl_6 \cdot 6H_2O$, $PdCl_2$, $HAuCl_4 \cdot xH_2O$, $H_2IrCl_6 \cdot 6H_2O$, $RhCl_3 \cdot xH_2O$, $RuCl_3 \cdot xH_2O$ were purchased from Aladdin Reagents Co., Ltd. $4MgCO_3 \cdot Mg(OH)_2 \cdot 5H_2O$ and pyridine (C_5H_5N) were supplied by Xilong Scientific Co., Ltd. γ-Al_2O_3, $Fe(NO_3)_3 \cdot 9H_2O$, $Co(NO_3)_2 \cdot 6H_2O$, $Zn(NO_3)_2 \cdot 6H_2O$, $CO(NH_2)_2$, 2-Methylimidazole, polyvinyl pyrrolidone, methanol (CH_3OH), ethanol (C_2H_5OH), 2-propanol (C_3H_8O), potassium hydroxide (KOH), hydrochloric acid (HCl) and sulfuric acid (H_2SO_4) were obtained from Sinopharm Chemical Reagent Co., Ltd. Graphene oxide (GO) dispersion solution was purchased from Shanghai Macklin Biochemical Co., Ltd. The 20 wt% Pt/C commercial catalyst was purchased from TANAKA, and 5 wt% Nafion was sourced from Dupont. All reagents and chemicals were used as received without further purification. The ultrapure water (18.25 MΩ cm) was used in all experiments.

Synthesis of M_1/hNCNC (M=Pd, Au, Ir, Rh, Ru) and HESAC

hNCNC (N: ~9.2 at%) was synthesized by *in-situ* MgO template method using pyridine as the carbon source [1]. The metal precursor solution was one of H_2PtCl_6, $PdCl_2$, $HAuCl_4$, H_2IrCl_6, $RhCl_3$ or $RuCl_3$ dissolved in HCl solution (pH=0~3). The metal precursors existed as coordination anions, i.e., $[PtCl_6]^{2-}$, $[PdCl_4]^{2-}$, $[AuCl_4]^{-}$, $[IrCl_6]^{2-}$, $[RhCl_6]^{3-}$, and $[RuCl_6]^{3-}$. The precursor solution (concentration: 0.001~0.2 mg mL^{-1}) and a homogeneous dispersion of hNCNC support (concentration: 0.1~1.5 mg mL^{-1}; dispersant: deionized water; stirring speed: 400 rpm) were co-pumped simultaneously at a set rate (5~20 mL min^{-1}) in the same direction, and uniformly mixed in a PTFE tube reactor at the preset temperature (50~80°C). The experimental conditions for the samples used in this paper are listed in Table S1. After stabilizing both the temperature and flow rate, the black suspension was collected at the reactor outlet. Then, the M_1/hNCNC catalysts were conveniently obtained after filtration, washing and drying (60°C).

The synthetic procedure for HESAC was similar to that for M_1/hNCNC. By replacing the single metal precursor solution with a mixed solution of $Co(NO_3)_2 \cdot 6H_2O$, $Ni(NO_3)_2 \cdot 6H_2O$, $Cr(NO_3)_3 \cdot 9H_2O$, and $Mn(NO_3)_2 \cdot 4H_2O$, the obtained product was denoted as HESAC-pre-1. HESAC-pre-1 was heated to 900°C at a ramping rate of 10°C min^{-1} under an Ar flow, and kept there for 1 h. The resulting product was immersed into 100 mL 2 mol L^{-1} H_2SO_4 solution to remove the metal nanoparticles, yielding the HESAC-pre-2. Finally, by using HESAC-pre-2 as the support and a mixed solution of H_2PtCl_6, $PdCl_2$, $HAuCl_4$, H_2IrCl_6, $RhCl_3$ and $RuCl_3$, the ten-element HESAC (PtPdAuIrRhRuCoNiCrMn) was prepared via the same continuous-flow synthesis method.

Synthesis of Pt_1/NCNT, Pt_1/g-C_3N_4, Pt_1/N-rGO and Pt_1/ZIF8-NC

The supports, including nitrogen-doped carbon nanotubes (NCNT), carbon nitride

nanosheets ($g-C_3N_4$), nitrogen-doped reduced graphene oxide (N-rGO) and ZIF8-derived nitrogen-doped carbon (NC) were synthesized following previously reported procedures [2–5]. Detailed synthesis protocols for each support are described as follows:

NCNT [2]: First, 2 g of γ-Al_2O_3 was dispersed in an appropriate volume of solution containing 2 mol L^{-1} $Fe(NO_3)_3$ and $Co(NO_3)_2$ via ultrasonic treatment. After freeze-drying, the resulting powder was placed into a quartz tube within a vertical furnace. The tube was heated to 650°C at a heating rate of 10°C min^{-1} under Ar flow (100 mL min^{-1}). Then pyridine was injected into the reactor at a rate of 100 μL min^{-1} for 8 min using a constant-flow pump. The furnace was held at 650°C for 15 min and then naturally cooled down to room temperature. The NCNT was obtained after sequential washing with 1 mol L^{-1} KOH and 6 mol L^{-1} HCl (to remove Al_2O_3 and Fe&Co species, respectively), followed by drying at 70°C for 12 h.

$g-C_3N_4$ [3]: 12 g of urea was placed into a covered alumina crucible, which was then heated at 600°C for 2 h in a muffle furnace. After natural cooling to room temperature, $g-C_3N_4$ nanosheets were collected directly.

N-rGO) [4]: N-rGO was synthesized by pyrolyzing a mixture of graphene oxide (GO) and urea. First, GO and urea (with a weight ratio of 1:5) were uniformly dispersed in deionized water by stirring and ultrasonic treatment, followed by freeze-drying. The obtained GO/urea composite powder was annealed in a tube furnace with a ramping rate of 10°C min^{-1} to 800°C and maintained at this temperature for 2 h under Ar flow (100 mL min^{-1}). After natural cooling, the N-rGO powder was collected and re-dispersed in ultrapure water prior to use.

ZIF8-derived NC [5]: First, 500 mg of m-$MgCO_3 \cdot Mg(OH)_2$ and 500 mg of polyvinyl pyrrolidone (PVP) were added into 100 mL methanol. The mixture was subjected to ultrasonic treatment for 30 min and vigorous stirring for 6 h. The PVP-modified m-$MgCO_3 \cdot Mg(OH)_2$ was collected by centrifugation and uniformly redispersed in 300 mL methanol. Subsequently, 850 mg of $Zn(NO_3)_2 \cdot 6H_2O$ and 1 g of 2-methylimidazole were added to the solution under vigorous stirring at 60°C for 12 h to form ZIF-8/PVP/m-$MgCO_3 \cdot Mg(OH)_2$ composites. After centrifugation, the solid precipitate was collected for pyrolysis with a ramping rate of 10°C min^{-1} to 900°C and kept for 2 h under Ar flow (100 mL min^{-1}). The ZIF8-NC product was obtained after repeated washing with 2 mol L^{-1} H_2SO_4 (to remove in situ generated MgO template and possible Zn species) and deionized water, followed by drying at 70°C for 12 h.

The synthetic procedure for Pt_1/NCNT, Pt_1/$g-C_3N_4$, Pt_1/N-rGO and Pt_1/ZIF8-NC was similar to that for Pt_1/hNCNC.

Characterization

The morphology of the as-prepared catalysts was observed by scanning electron microscopy (SEM, Hitachi, Regulus 8100), high-resolution transmission electron microscopy (HRTEM, JEOL, JEM-2100) and high-angle annular dark-field scanning transmission electron microscope (HAADF-STEM, Thermo Fisher Scientific, FEI Titan Themis 60-300). The

composition and crystal structure were characterized by X-ray diffraction (XRD, Bruker D8 Advance A25, Cu-K_α radiation of 1.5406 Å), X-ray photoelectron spectroscopy (XPS, ULVAC-PHI INC, PHI 5000 VersaProbe, Al K_α), and Laser confocal Raman spectroscopy (Raman, LabRAM Aramis, laser excitation wavelength of 532 nm). X-ray absorption fine structure (XAFS) was carried out at the BL11B beamline of the Shanghai Synchrotron Radiation Facility (SSRF). XANES and EXAFS data reduction and analysis were performed using the IFEFFIT package with Athena software. The metal content was determined by inductively coupled plasma optical emission spectrometry (ICP-OES, PerkinElmer, Avio500), ultraviolet visible spectroscopy (UV-vis, Shimadzu UV-3600), and thermogravimetric analysis (TG, Netzsch STA449F3). Brunauer-Emmett-Teller (BET) surface area was measured on a Thermo Fisher Scientific Surface Gas Adsorption Porosimeter at liquid nitrogen temperature with pre-evacuation at 300°C for 6 h. The specific surface area was estimated based on the BET method using adsorption data in the relative pressure (p/p_0) range 0.08~0.3. Pore size distributions were derived from adsorption data using the Hovarth-Kawazoe method (for micropores <2 nm) and the Barret-Joyner-Halenda (BJH) method (for mesopores and macropores >2 nm). The bulk conductivity of the supports and catalysts was measured by four-wire method using a source measure unit (Keithley 6430) [6].

Electrochemical measurements in a three-electrode system

Electrochemical measurements were carried out on an electrochemical workstation (CHI 760E, Shanghai Chenhua Instrument Co., Ltd., China) using a standard three-electrode configuration. The working electrode was a catalyst-modified glassy carbon (GC) electrode, the counter electrode a high-purity graphite rod, and the reference electrode an Ag/AgCl electrode (KCl saturated). The electrolyte was 0.5 M H_2SO_4 aqueous solution bubbled with N_2 to remove dissolved oxygen. The catalyst ink was prepared by ultrasonically dispersing a mixture consisting of 2 mg of catalyst, 750 μL of ultrapure water, 200 μL of ethanol, and 50 μL of 5 wt% Nafion solution. 10 μL of the catalyst ink was spread onto the surface of the GC electrode (geometric area: 0.196 cm^2) and dried in air at room temperature. For evaluating the HER activity, cyclic voltammograms (CVs) were recorded at a scan rate of 100 mV s^{-1}, and linear sweep voltammograms (LSVs) at 5 mV s^{-1}, unless otherwise specified. Tafel slopes were derived from the LSV curves using the Tafel equation ($\eta = a + b \log j$), where η is overpotential, a is Tafel intercept, b is Tafel slope, and j is current density). Electrochemical impedance spectroscopy (EIS) was conducted over a frequency range of 100 kHz to 0.01 Hz with an amplitude of 5 mV. All potentials were calibrated to the reversible hydrogen electrode (RHE) using the following equation: $E_{RHE} = E_{Ag/AgCl} + 0.209 + 0.0591 \times pH$. All electrochemical tests were performed with 95% iR compensation enabled via the CHI workstation software.

Electrochemical measurements in PEMWE.

To construct the membrane electrode assembly (MEA), Pt_1/hNCNC or commercial Pt/C (60 wt%) was employed as cathode catalyst, while commercial served IrO_2 as anode catalyst [7]. To prepare the anode and cathode inks, the corresponding catalysts were dispersed in a mixture of isopropanol, deionized water and 5 wt% Nafion solution. The mass ratio of ionomer Nafion to catalyst was set to 1:3. After ultrasonic dispersion in an ice water for at least 1 h, a homogeneous catalyst ink was obtained. An ultrasonic spray technique was adopted to deposit the catalyst inks onto a Nafion 115 membrane (Dupont), forming a catalyst-coated membrane (CCM). The anode loading of IrO_2 was 1.5 mg cm^{-2}. The cathode loading of Pt_1/hNCNC was 20 μg_{Pt} cm^{-2}, and that of commercial Pt/C (60 wt%) was 20 or 100 μg_{Pt} cm^{-2} for comparison. Commercial Ti fiber felt was directly used as the gas diffusion layer (GDL). The PEMWE was operated at 80°C, with the deionized water fed as reactant at a flow rate of 20 mL min^{-1}. Before testing, the PEMWE was activated at 1.0 A cm^{-2} for 1 h. The durability of the PEMWE was evaluated via chronopotentiometry at 1.0 A cm^{-2} under the same operating conditions. All presented data were recorded without *iR* compensation.

Theoretical calculations

The density functional theory (DFT) calculations: Free energy profiles were calculated using DFT package of $DMol^3$ in Materials Studio (Accelrys, Inc) (Figs. S11, S12) [8]. The exchange-correlation interaction was described within the generalized gradient approximation (GGA) expressed by the Perdew-Burke-Ernzerhof (PBE) functional [9]. A double numerical basis with polarization functions (DNP) was adopted, with a global orbital cutoff of 5.0 Å. The conductor-like screening model (COSMO) was utilized to incorporate the implicit solvent effect, with a dielectric constant (ε) of 78.54 to represent water [10]. The self-consistent field (SCF) convergence threshold was set to be 10^{-6} eV. For structural optimization, the tolerances for energy, force and displacement were set to 1×10^{-6} Ha, 0.002 Ha $Å^{-1}$ and 5×10^{-3}Å, respectively. A Monkhorst-Pack k-point mesh of 4×4×1 was applied to sample the first Brillouin zone. The graphene structure was constructed based on a $p(8 \times 8 \times 1)$ supercell of single-layer graphite. Models of M_1/hNCNC (M=Pt, Pd, Au, Ir, Rh, Ru) were constructed by introducing a 6Å-diameter hole into graphene, with a single M atom bonded with N or C at the hole edge. A vacuum layer of 25 Å was introduced to avoid interactions between periodic images. Atomic charges were obtained via Mulliken's scheme [11,12]. The free energy diagrams were calculated with the method elaborated in our previous study [13].

Ab initio molecular dynamics (AIMD) simulations: The dynamic attachment of free-moving $[PtCl_6]^{2-}$ on hNCNC were conducted by AIMD simulations (Fig. 3e,f). A graphene layer with pyridinic-N dopants and micropores was constructed, and a $[PtCl_6]^{2-}$ precursor was positioned outside the layer as the initial configuration. The model dimensions are 17.04 × 19.68 × 23.40 $Å^3$. Calculations were performed using the CP2K 2024.1 package [14,15], with the PBE-D3(BJ) functional to describe exchange-correlation and dispersion interactions [16,17].

Goedecker-Teter-Hutter (GTH) pseudopotentials and DZVP-MOLOPT-SR-GTH basis sets were utilized [18,19]. The planewave expansion cutoff energy was set to 600 Ry, and the time step was 1.0 fs. The SCF cycles employed the orbital transformation (OT) algorithm. Under the canonical ensemble (NVT ensemble), a Nosé-Hoover thermostat was used with a target temperature of 350 K [20,21]. A total trajectory length of 6 ps was integrated. After a 2 ps equilibration period, $[PtCl_6]^{2-}$ rapidly diffused into the micropores. During the simulation, energy and atomic coordinates were recorded every 10 fs.

Molecular dynamics (MD) simulations: MD simulations of mixing models were performed using the August, 2025 version of LAMMPS (Fig. 3a-c) [22], operating in two dimensions with real units and a time step of 0.5 fs. The mobile fluid was maintained at 300 K using a Langevin thermostat with a damping coefficient of 100 fs, while all wall atoms were fixed to preserve structural integrity. Two mixing modes were investigated: direct batch mixing and continuous-flow mixing in a Y-shaped channel. The direct batch mixing model consists of a 50 Å × 120 Å rectangular cell, periodic along the x-axis and closed along the y-axis. For the continuous-flow system, a Y-shaped channel was constructed with a height of 1500 Å, wall thickness of 0.5 Å, and uniform channel width of 6 Å. Each system comprises 100 hNCNC particles (represented as red spheres) and 500 $[PtCl_6]^{2-}$ ions (represented as blue particles). In the direct batch mixing model, the hNCNC was initialized in the upper region, whereas $[PtCl_6]^{2-}$ ions were placed in the lower reservoir. In the Y-channel mode, the two species were introduced through separated branches. Initial positions were randomized to ensure an unbiased starting distribution. To drive spontaneous mixing, a constant gravitational acceleration was applied along the y-axis. The Lennard-Jones 12-6 potential was used as the force field. When a $[PtCl_6]^{2-}$ ion appeared within the first-neighbor coordination shell of hNCNC particle, defined as 2 Å in thickness, the ion was captured and recorded. The saturation adsorption capacity for each hNCNC particle was set as 10 $[PtCl_6]^{2-}$ ions. At fixed time intervals (every 500 MD steps), each hNCNC particle irreversibly adsorbs all $[PtCl_6]^{2-}$ ions appeared within its first-neighbor coordination shell, mimicking diffusion-limited capture. Over time, the $[PtCl_6]^{2-}$ ions depleted in solution and the corresponding adsorption capacities per hNCNC particle were monitored to evaluate mixing uniformity and adsorption efficiency. To enhance statistical reliability and reduce stochastic fluctuations in MD simulations, 12 independent simulations with different random seeds were performed, yielding a clear statistical distribution of adsorption behavior (Fig. 3c).

Finite element method (FEM) simulations: The concentration distribution of H_2PtCl_6 around hNCNC module was simulated using a FEM solver. Based on the Nano-CT images of hNCNC, we reconstructed a 3D microelement model (cross-sectional area: 0.7 mm × 0.4 mm, thickness: 1 mm) with a minimum mesh size of 0.0225 mm, allowing for precise three-dimensional mesh modeling (Fig. S10). The simulation used three modules: Transport of Diluted Species, Laminar Flow, and General Form PDE (partial differential equation). In the Transport of Diluted Species module, the external concentration at the left boundary and the

initial bulk concentration of $[PtCl_6]^{2-}$ ions were both set as c_0, and the right boundary was set as the outlet. The remaining boundaries (up, bottom, front, back) were assigned periodic conditions. An adsorption flux was added to the hNCNC surface as a source term. Accordingly, the Nernst–Planck equation governing the mass transport process on the hNCNC surface follows the following equation [23]:

$$\frac{\partial c}{\partial t} + \nabla \cdot (-D\nabla c) + \boldsymbol{u} \cdot \nabla c = -k_{\mathrm{des}} c_{\mathrm{s}} + k_{\mathrm{ads}} c(\mathit{\Gamma}_{\mathrm{s}} - c_{\mathrm{s}}) \tag{1}$$

Where c and c_{s} is the bulk and surface concentration of $[PtCl_6]^{2-}$, respectively; D is the bulk diffusion coefficient of $[PtCl_6]^{2-}$; k_{ads} and k_{des} are the adsorption and desorption rate constants, respectively; and $\mathit{\Gamma}_{\mathrm{s}}$ is the saturation adsorption site density (Table S3).

The mass transport process was coupled with the Laminar Flow module to define the properties of fluid viscosity and density, initial velocity distribution of flow field, and boundary conditions of solid walls. Periodic boundary conditions and pressure point constraints were applied to the flow field to simulate fluid flow around the hNCNC module. The adsorption process was implemented on the hNCNC surface and described by the following PDE [24]:

$$\frac{\partial c_s}{\partial t} + \nabla \cdot (-D_{\mathrm{s}} \nabla c_{\mathrm{s}}) = k_{\mathrm{ads}} c(\mathit{\Gamma}_{\mathrm{s}} - c_{\mathrm{s}}) - k_{\mathrm{des}} c_{\mathrm{s}} \tag{2}$$

here D_{s} is the surface diffusion coefficient of $[PtCl_6]^{2-}$ (Table S3).

Based on the above simulations, we obtained the outlet concentration within the microelement, $c_{\mathrm{out}}(t, c_{\mathrm{in}})$, as a function of adsorption time and initial concentration, which was fitted using Python Script (Fig. S10f). In the spiral flow reactor, the adsorption process continuously accumulates. By iteratively applying the derived function relationship $c_{\mathrm{out}} = \mathrm{f}(c_{\mathrm{in}})$, we calculated the local $[PtCl_6]^{2-}$ concentration at various points within the reactor (Fig. 3d).

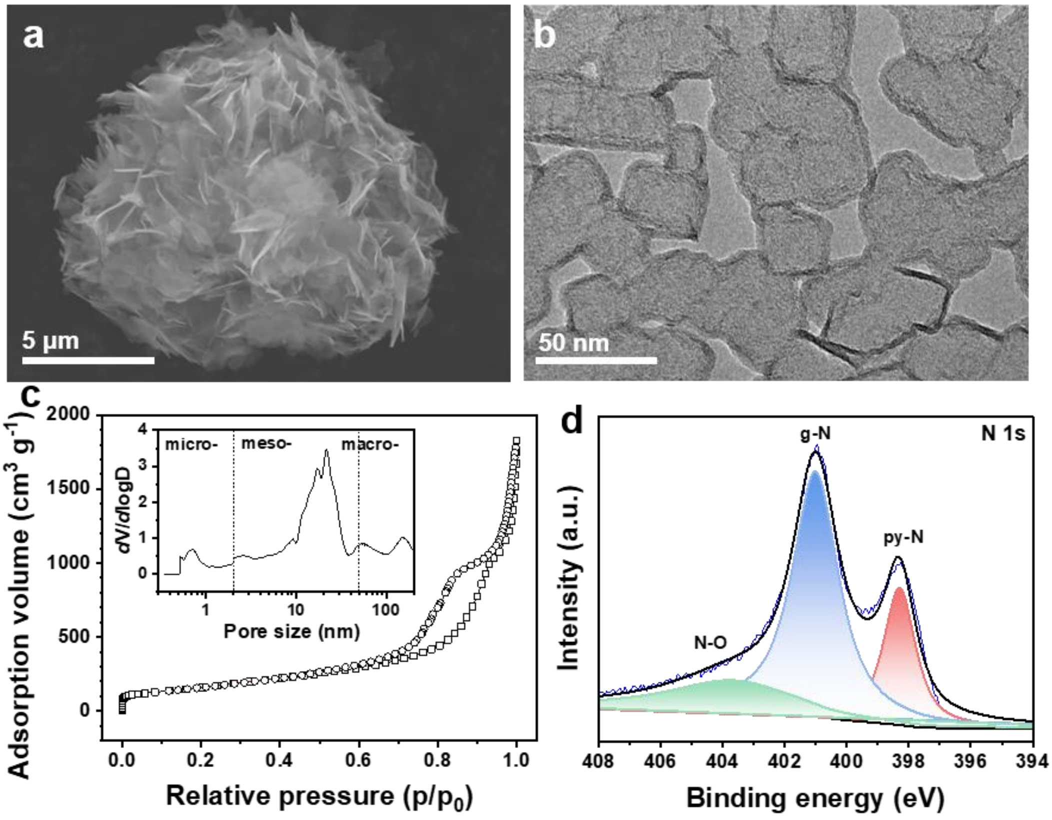

Figure S1. Morphology and structure characterizations of hNCNC support. (a) SEM image. (b) TEM image. (c) N_2 adsorption-desorption isotherm. Inset is the corresponding pore size distribution. (d) N 1s fine XPS spectrum.

The hNCNC has a coexisting micro-, meso-, and macro-porous structure with a high specific surface area of 611 m^2 g^{-1}. Abundant micropores (~0.6 nm in size) are distributed across the carbon shell, favourable to the anchoring of Pt single atoms. [25]. The conductivity of hNCNC was ~1480 S m^{-1}, as measured by the four-wire method [6]. XPS analysis reveals the chemical composition of C (86.8 at.%), N (9.2 at.%) and O (4.0 at.%), respectively. The N 1s spectrum is composed of signals of pyridinic-N (~398.3 eV), graphitic-N (~401.0 eV) and oxidized-N (~403.6 eV) (Fig. S1d) [26].

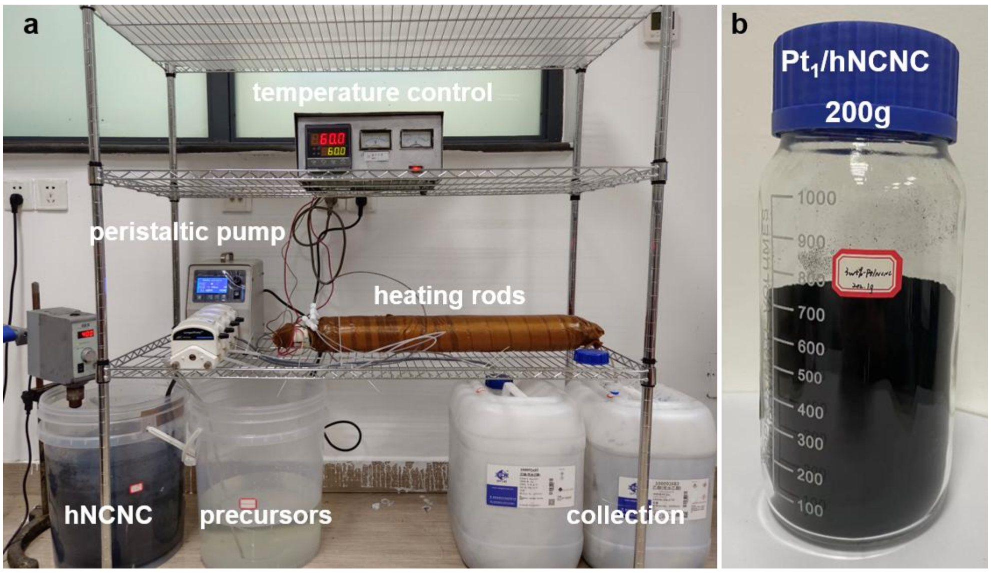

Figure S2. (a) Photograph of the home-made microfluidic reactor. (b) Photograph of 200 g sample of 3 wt%-Pt_1/hNCNC.

In a typical case, the flow microfluidic reactor operated at a flow rate of 15 mL min^{-1} (support concentration: 1.5 mg mL^{-1}, see Table S1), which delivers a productivity of ~1350 mg h^{-1}, about 321 times higher than that of the batch synthesis (Fig. S3).

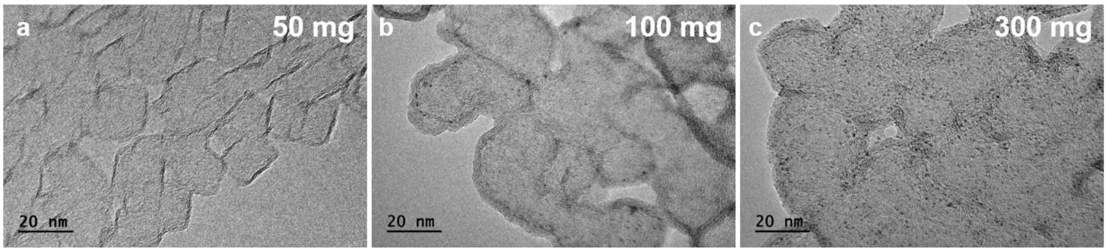

Figure S3. TEM image of Pt/hNCNC synthesized via flask-scale impregnation-adsorption, with single-batch yields of 50 mg (a),100 mg (b), and 300 mg (c).

Batch impregnation-adsorption method was used to prepare the 5 wt%-Pt/hNCNC samples with 50, 100, and 300 mg single-batch yields, with a typical operation time of 12 h. High-quality Pt_1/hNCNC SAC was obtained at 50 mg, corresponding to a productivity of ~4.2 mg h^{-1}[13]. A small number of Pt clusters appeared at 100 mg, while both the number and size of Pt clusters significantly increased at 300 mg.

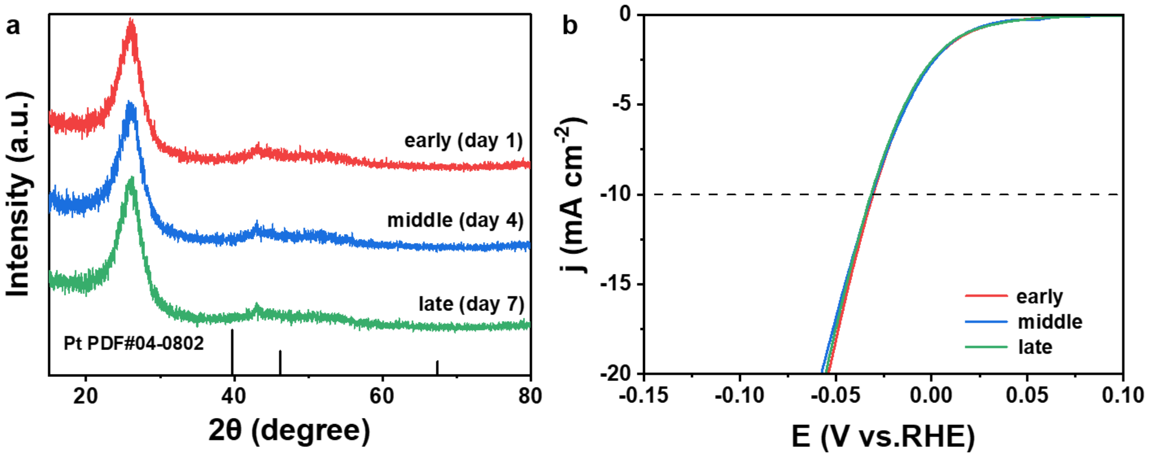

Figure S4. Pt_1/hNCNC collected at different stages during the continuous-flow synthesis. (a) XRD patterns. (b) LSV curves measured in 0.5 M H_2SO_4 solution.

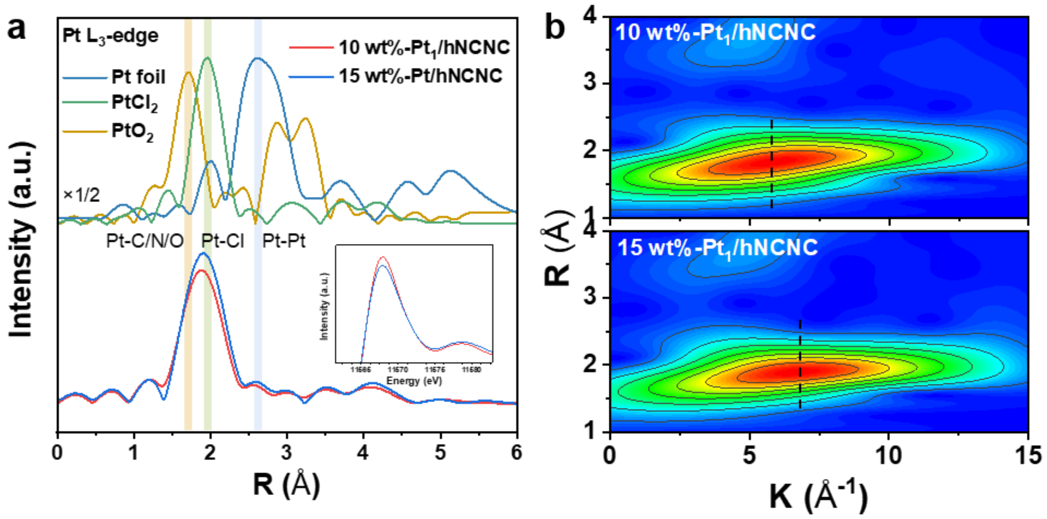

Figure S5. XAFS characterizations of 15 wt%-Pt/hNCNC. (a) k^2-weighted R-space FT-EXAFS. Inset is normalized Pt L_3-edge XANES spectra. (b) WT-EXAFS of 15 wt%-Pt/hNCNC. The data for 10 wt%-Pt_1/hNCNC are also showned for comparison.

For 15 wt%-Pt/hNCNC, XANES spectra reveal a slightly lower oxidation state compared to the 10 wt%-Pt_1/hNCNC sample. Little Pt-Pt peak at ~2.61 Å confirms the dominant atomic dispersion of Pt with a small fraction of sub-nm clusters, consistent with HAADF-STEM observations (Fig. 2c and Fig. S5a). The rightward shift of the K-space center arises from scattering signals of trace Pt-Pt clusters in 15 wt%-Pt/hNCNC (Fig. S5b).

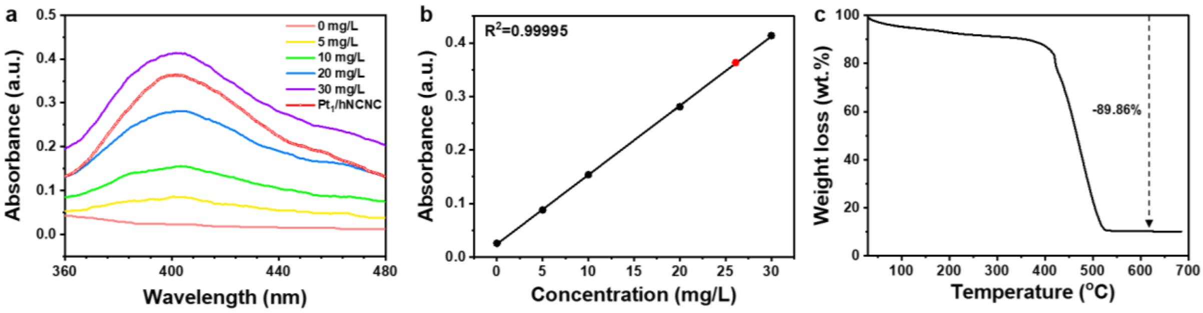

Figure S6. Measurements of Pt loading in 10 wt%-Pt_1/hNCNC. (a,b) UV-vis absorption spectra (a) and corresponding working plot (b); (c) TG curve.

Pt content was measured to be ca. 9.66 wt% by UV-vis absorption tests (Fig. S6a,b) [7]. The TG analysis shows that the Pt weight fraction is ca. 10.14 wt% (Fig. S6c).

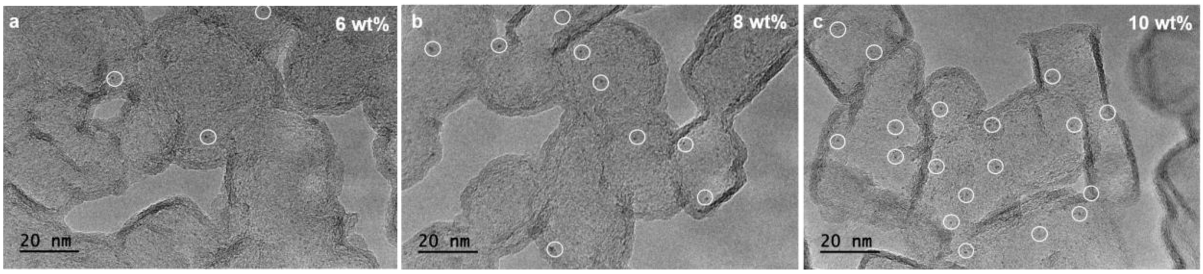

Figure S7. TEM images of Pt/hNCNC catalysts obtained by the flask-scale impregnation-adsorption method. (a) 6 wt%; (b) 8 wt%; (c) 10 wt%.

Pt nanoclusters/nanoparticles were often observed when Pt loading ≥ 6 wt%, as highlighted by white circles.

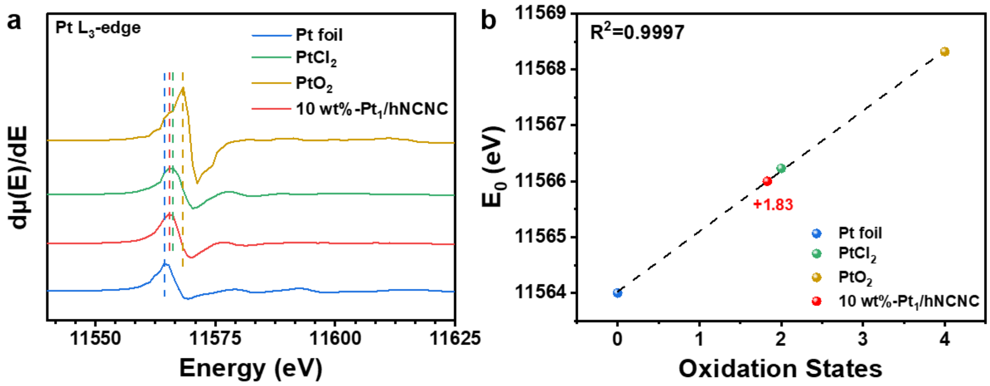

Figure S8. Analysis of the Pt oxidation state in 10 wt%-Pt_1/hNCNC. (a) First derivative spectrum of XANES. The spectra of Pt foil, $PtCl_2$ and PtO_2 are presented as the references. (b) The fitted average oxidation state of Pt in 10 wt%-Pt_1/hNCNC.

Based on the established correlation between absorption edge energy and Pt oxidation states, the average Pt valence is estimated to be *ca.* +1.83.

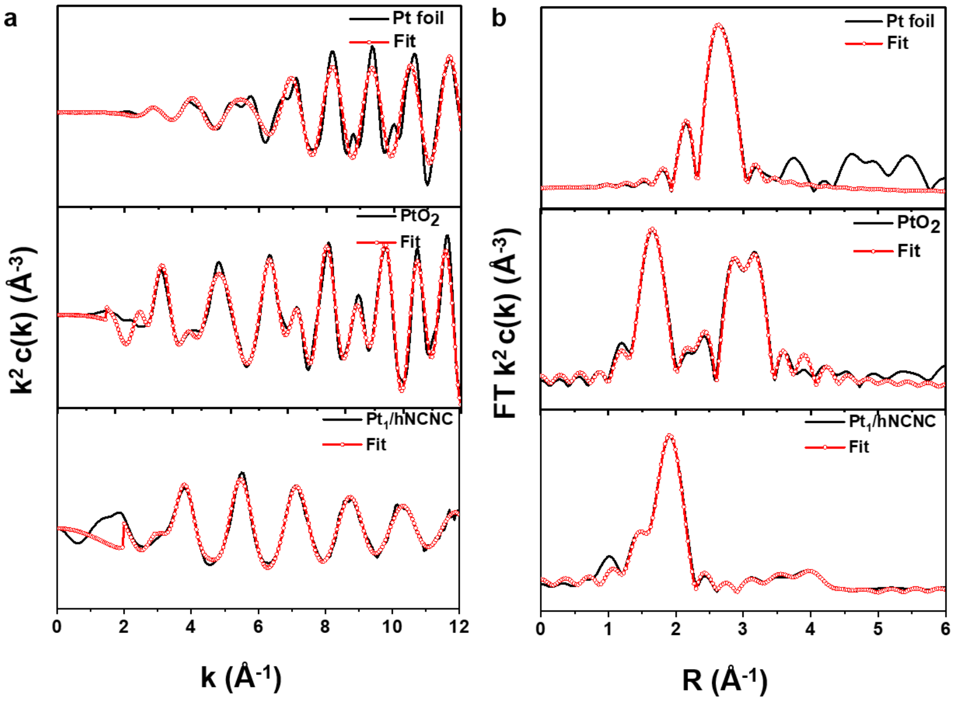

Figure S9. FT-EXAFS fitting curves of Pt foil, PtO_2 and Pt_1/hNCNC. (a) k^2 weighted k-space. (b) k^2 weighted R-space.

EXAFS fitting evidences a well match between measured and calculated spectra. The fitting parameters are listed in Table S2.

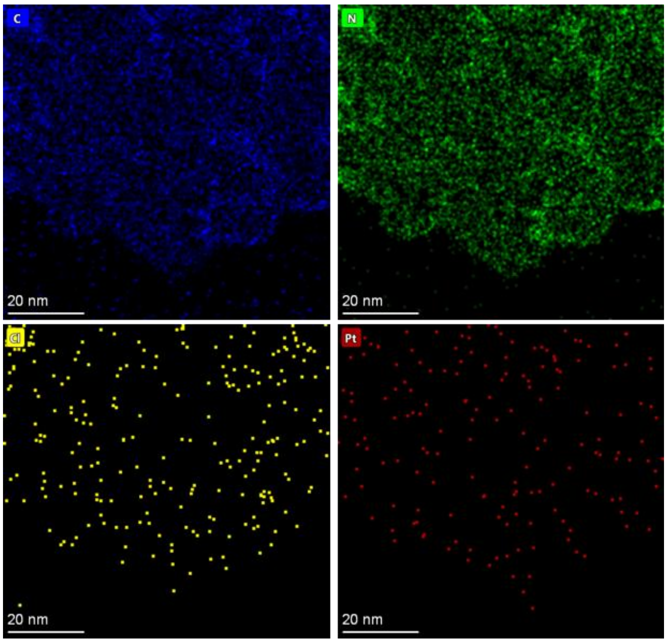

Figure S10. Homogeneous spatial distributions of C, N, Cl, and Pt for 10 wt%-Pt_1/hNCNC observed by Energy-dispersive X-ray spectroscopy elemental mapping.

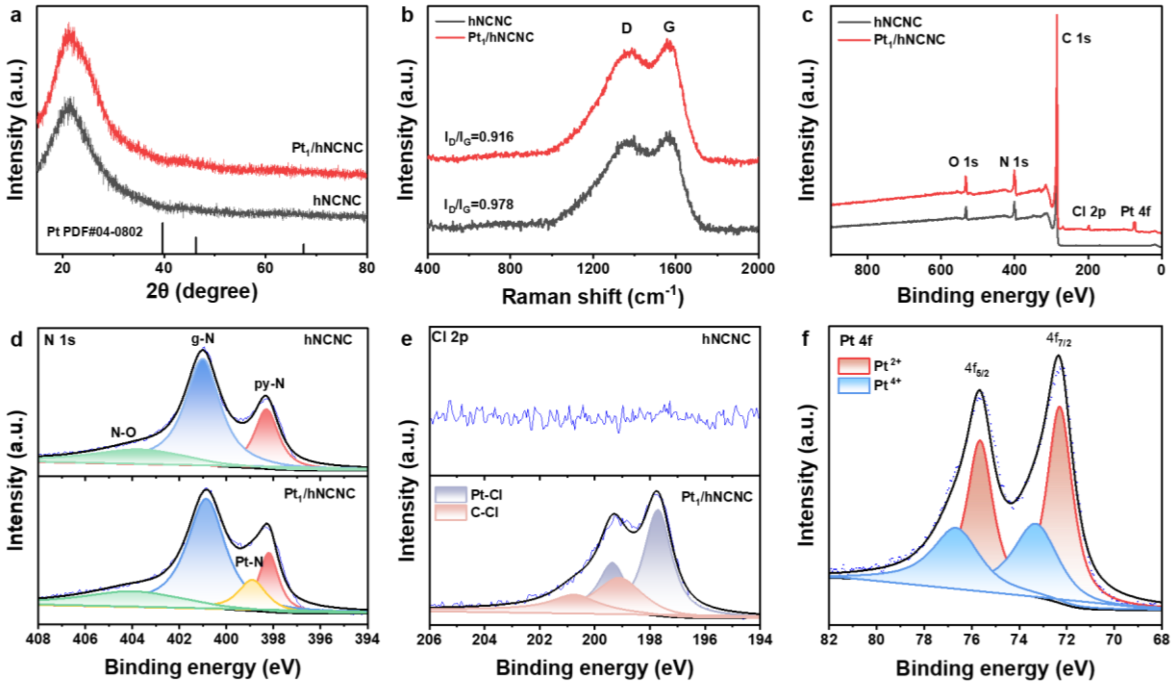

Figure S11. Characterizations on 10 wt%-Pt_1/hNCNC and hNCNC support. (a) XRD patterns. (b) Raman spectra. (c) XPS survey spectra. (d-f) N 1s (d), Cl 2p (e) and Pt 4f (f) fine spectra.

XRD pattern displays only the graphite (002) peak at ~26°without detectable Pt crystallite signals (Fig. S11a) [27]. After anchoring Pt single atoms, the I_D/I_G ratio in Raman spectra decreases from 0.978 to 0.916, indicating efficient utilization of micropores for Pt single-atom immobilization (Fig. S11b) [28]. XPS analysis reveals the dominant Pt^{2+} ($4f_{7/2}$: 72.45 eV, $4f_{5/2}$: 75.80 eV) and minor Pt^{4+} (73.60, 76.95 eV) with the Pt content of 0.48 at% [29], accompanied by a low residual Cl content of ca. 0.97 at% mainly associated with Pt-Cl bonds (Fig. S11c-f) [30]. The atomic ratio of Pt/Cl in 10 wt%-Pt_1/hNCNC is *ca.* 1:2, consistent with the Pt-N_2Cl_2 coordination.

Based on the XPS analysis, the total N content in hNCNC is ~9.2 at%, with pyridinic-N accounting for ca. 25% (Fig. S1c). If we conservatively assume that two pyridinic-N sites are required to capture one Pt atom (forming Pt-N_2Cl_2 configuration), the theoretical Pt atomic percentage is estimated to be ~1.15 at%. This translates to a theoretical maximum Pt mass loading of ~15 wt%, which comfortably accommodates our experimentally achieved high loading up to 10 wt% while maintaining single-atom dispersion.

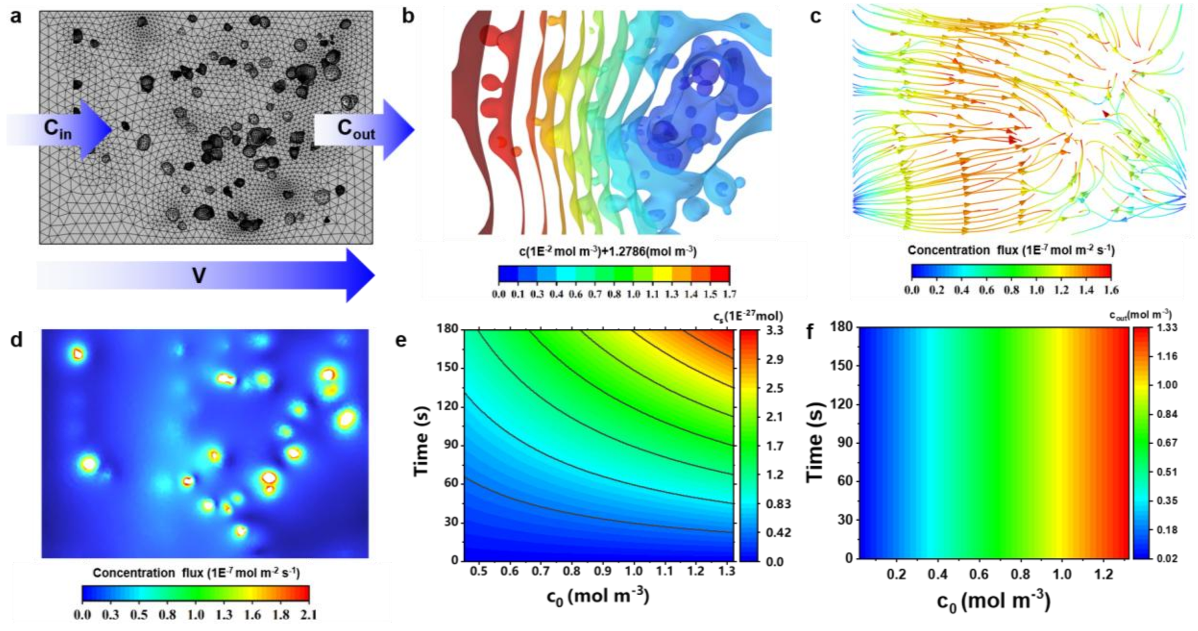

Figure S12. FEM simulation of continuous-flow synthesis. (a) 3D microelement model containing the hNCNC particles inside microfluidic reactor (Cross-sectional area: 0.7 mm × 0.4 mm, thickness: 1 mm). (b) Concentration isosurface plot. (c) Concentration flux streamline distribution. (d) Cloud map of concentration flux distribution at half of the cross-section. (e,f) Spatiotemporal evolution of $[PtCl_6]^{2-}$ concentration on hNCNC surface (e) or at the outlet (f) as a function of time and initial bulk concentration.

3D microelement model containing the hNCNC particles inside microfluidic reactor was reconstructed based on typical Nano-CT image, with a minimum mesh size of 0.0225 mm (Fig. S12a, Table S3). The concentration isosurface results show that the residual concentration of $[PtCl_6]^{2-}$ near hNCNC is significantly lower than that in the bulk solution (Fig. S12b). This effect is particularly pronounced around hNCNC with larger particle sizes, which exhibit broader low-ion-concentration regions and thus indicate a strong $[PtCl_6]^{2-}$ ions adsorption capability of the hNCNC surface. The concentration flux streamline distribution and cloud map further clearly illustrate that the majority of ion flux occurs near hNCNC surfaces, emphasizing this surface driven depletion mechanism (Fig. S12c, d). Based on the above simulations, we obtained the ions concentration at the outlet and on the hNCNC surface within a microelement, as the function of adsorption time and initial concentration (c_0) (Fig. S12e, f). In the spiral flow reactor, the adsorption process continuously accumulates. By iteratively applying the derived function relationship $c_{\text{out}} = \text{f}(c_{\text{in}})$, the fitted curve delineates the distribution of $[PtCl_6]^{2-}$ ion concentrations inside the reactor (Fig. 3d), serving as a reference for reactor design. This macroscopic perspective reveals the rapid adsorption kinetics and high adsorption capacity of hNCNC, which significantly influence the dispersion of Pt single atoms.

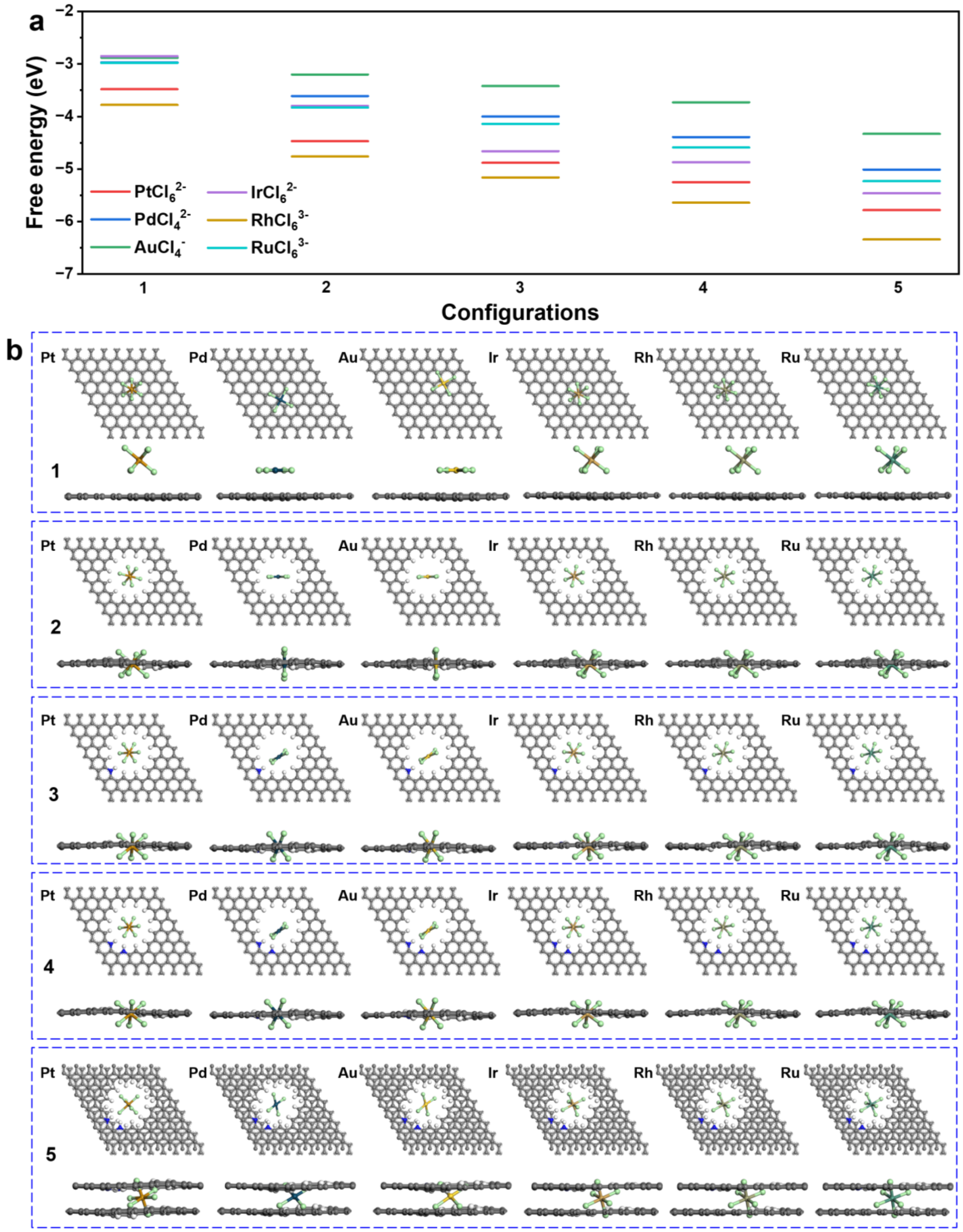

Figure S13. DFT calculations. (a) Free energy of different anions on five typical models. **1** Graphite sheet without micropore; **2-4** Graphitic mono-layer with a micropore (0.6 nm), decorated by 0, 1, 2 pyridinic-N atoms, respectively; **5** Graphitic bi-layer with a micropore (0.6 nm) decorated by 2 pyridinic-N atoms. (b) Corresponding configurations.

Adsorption of all anions is a spontaneous exothermic process. The adsorption energy (E_{ab}) is gradually reduced from model 1 to model 5 (Fig. S13a, Table S4).

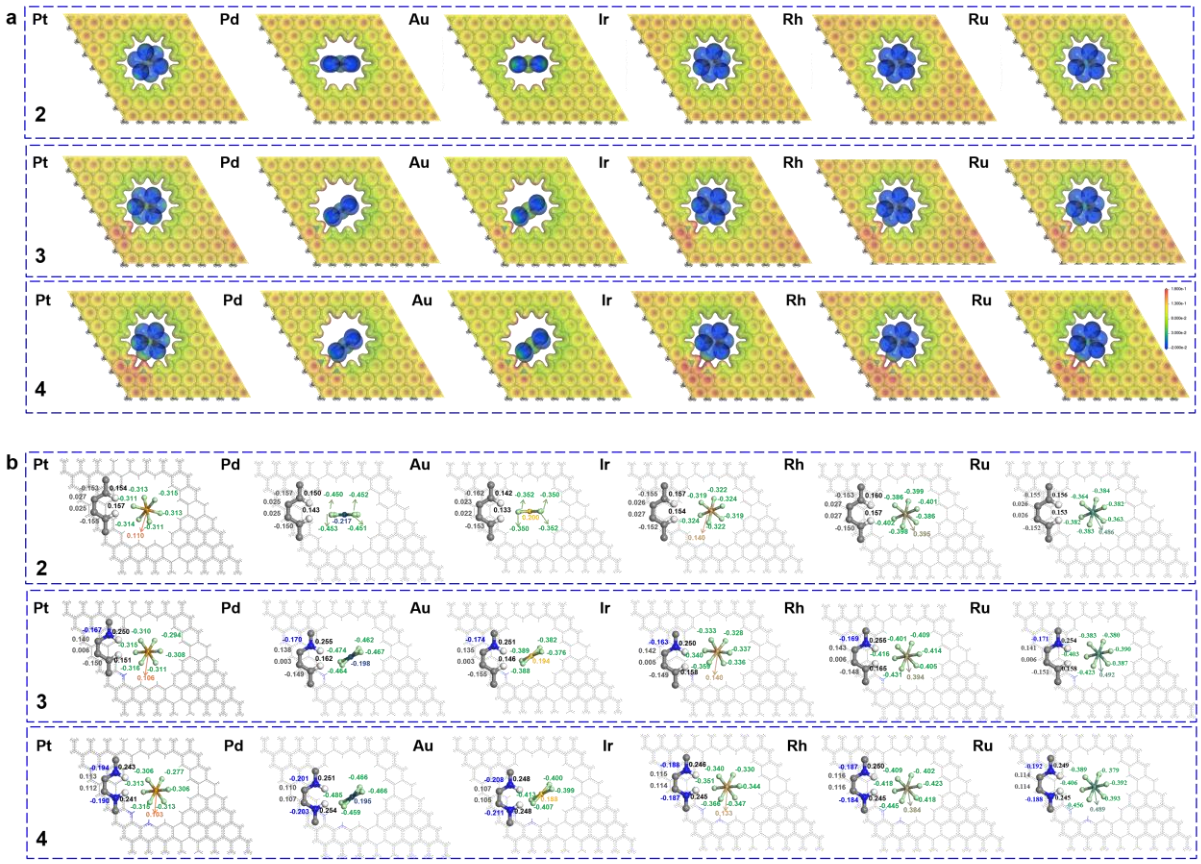

Figure S14. (a) Electrostatic potential surface and (b) charge population analysis of different anions inside graphitic mono-layer with the micropore decorated by 0, 1 and 2 pyridinic-N atoms (Models 2-4 in Fig. S13). Note: blue and red color in (a) represent electron accumulation and depletion.

Without N-doping (model 2), there is little red region around the pore with low positive charge. For the N-doped cases (models 3, 4), protonated pyridinic-N under acidic condition displays corresponding red region, forming distinct charge complementarity with the blue region on metal anions (Fig. S14a). The high positive-charge on the H atom bonded with N indicates strong electrostatic interaction with metal anions associated with enhanced capturing effect (Fig. S14b).

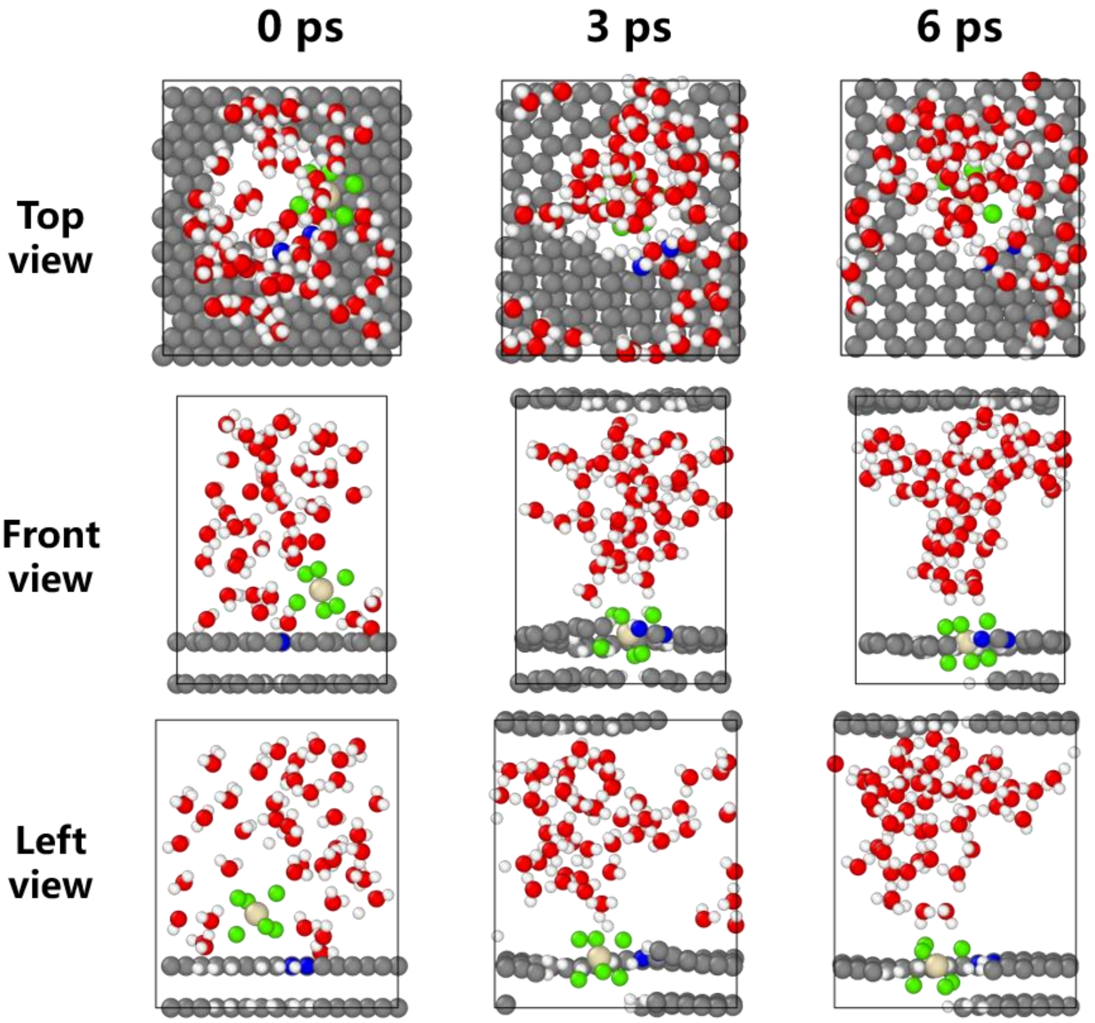

Figure S15. Structural snapshots of the system at 0, 3, and 6 ps, visualized from the top, front, and left views.

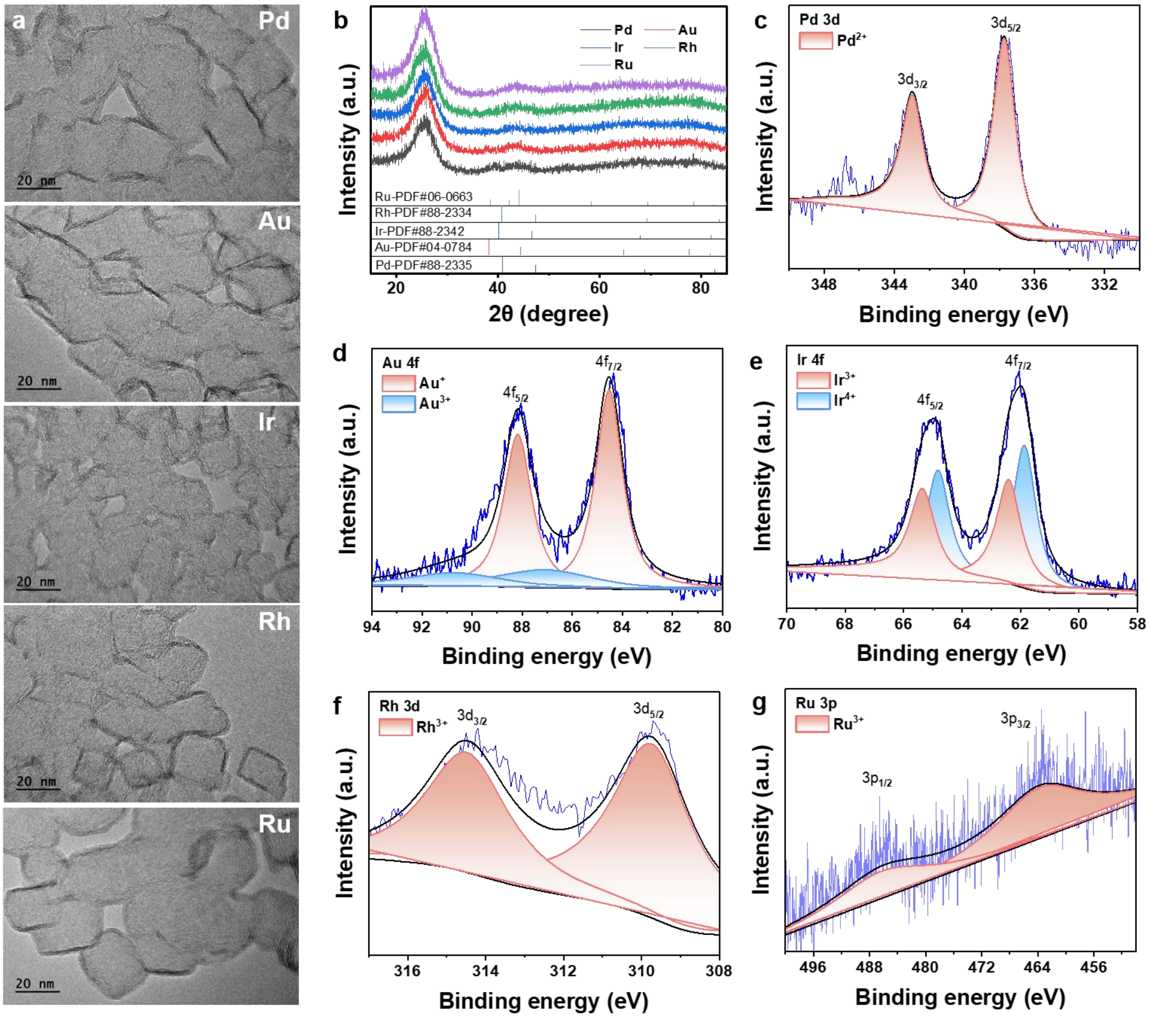

Figure S16. Morphology and structure characterizations of M_1/hNCNC (M=Pd, Au, Ir, Rh, Ru). (a) TEM images. (b) XRD patterns. (c-g) XPS fine spectra of Pd 3d (c), Au 4f (d), Ir 4f (e), Rh 3d (f), Ru 3p (g).

TEM and XRD characterizations confirm the absence of NPs and metal diffraction peaks in all samples (Fig. S16a,b). XPS analysis indicates the oxidation states of metal species, with characteristic binding energies at 337.75 eV (Pd $3d_{5/2}$), 84.40 eV (Au $4f_{7/2}$), 62.10 eV (Ir $4f_{7/2}$), 309.85 eV (Rh $3d_{5/2}$), and 463.0 eV (Ru $3p_{3/2}$), respectively (Fig. S16c) [31–35].

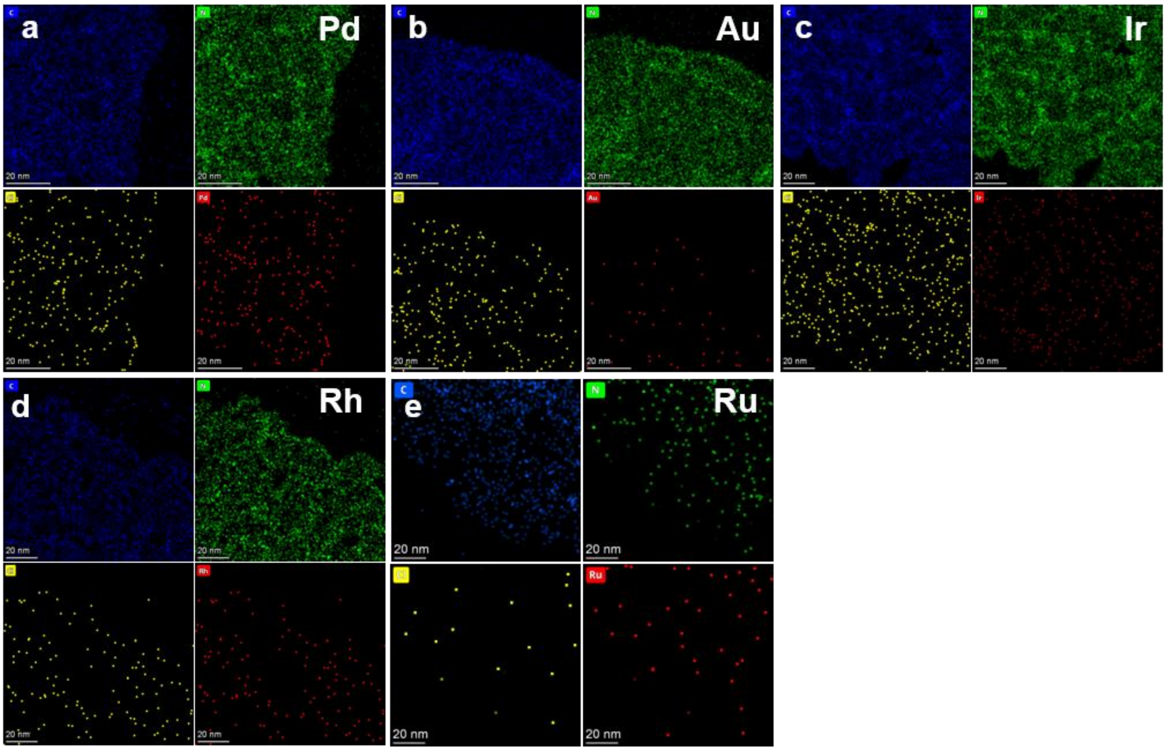

Figure S17. EDS elemental mapping images for M_1/hNCNC (M=Pd, Au, Ir, Rh, Ru).

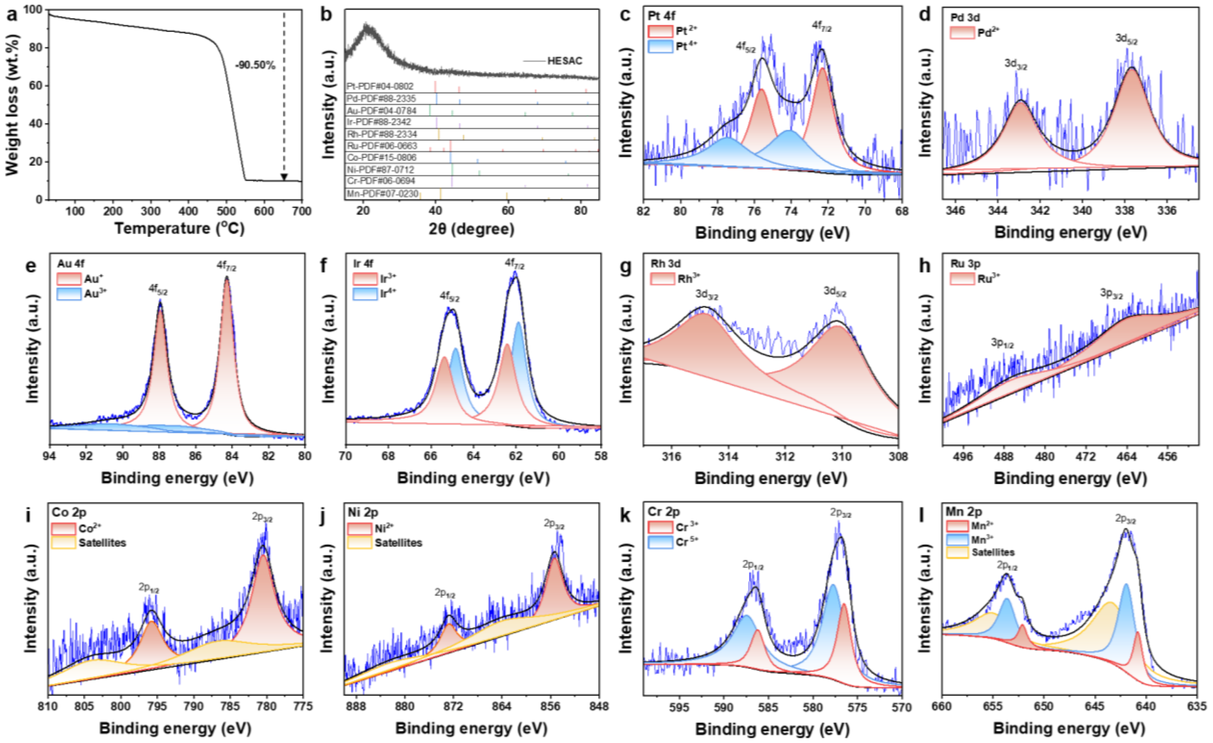

Figure S18. Structure characterizations of HESAC (M= Pt, Pd, Au, Ir, Rh, Ru, Co, Ni, Cr, and Mn). (a) TG curve. (b) XRD patterns. (c-l) XPS fine spectra of Pt 4f (c), Pd 3d (d), Au 4f (e), Ir 4f (f), Rh 3d (g), Ru 3p (h), Co 2p (i), Ni 2p (j) , Cr 2p (k), Mn 2p (l).

The total metal element content is ca. 9.5 wt% (Fig. S18a). No metal-based phases are detected by XRD analysis (Fig. S18b). XPS analysis indicates the positive oxidation states of all metal species (Fig. S18c-l).

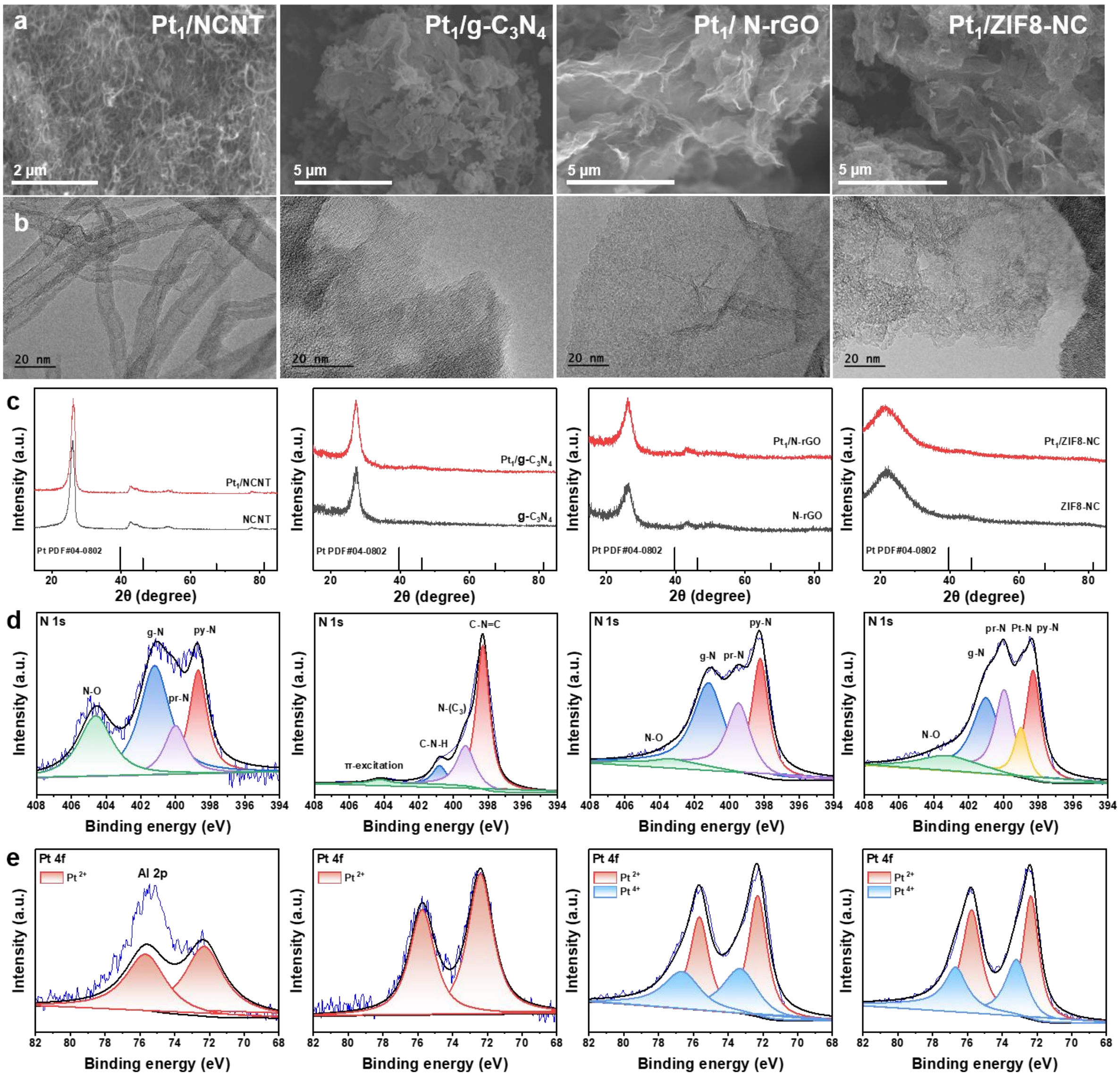

Figure S19. Morphology and structure characterizations of Pt_1/NCNT, Pt_1/g-C_3N_4, Pt_1/N-rGO and Pt_1/ZIF8-NC. (a) SEM images. (b) TEM images. (c) XRD patterns. (d) The XPS fine spectra of N 1s. (e) The XPS fine spectra of Pt 4f. Note: Al 2p came from the unremoved γ-Al_2O_3 in NCNT.

These nitrogen-doped carbon (NC) supports exhibit distinct morphologies (Fig. S19a,b). TEM and XRD verify the absence of Pt NPs in these Pt_1/NC catalysts, and XPS spectra indicate the oxidation state of Pt species (Fig. S19c-e).

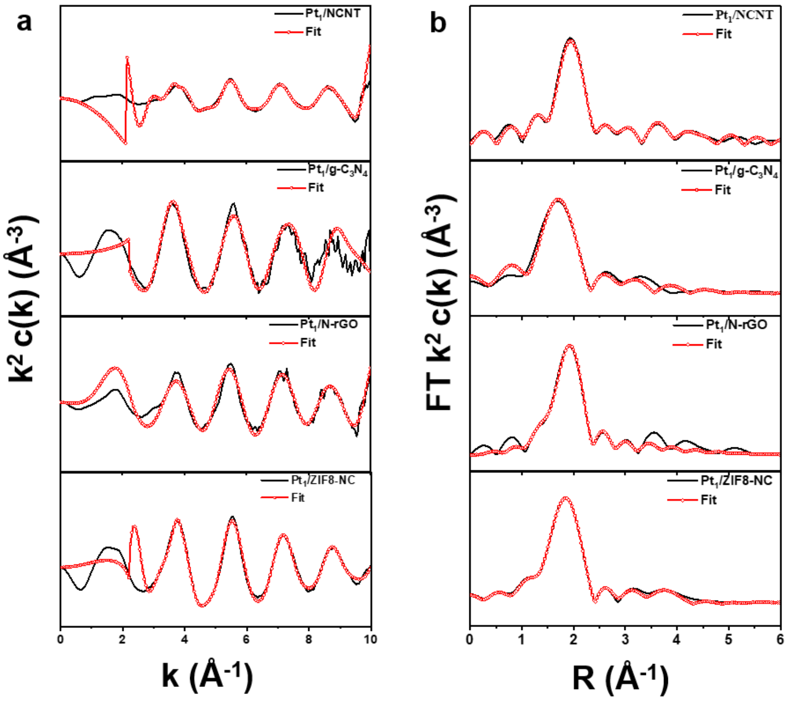

Figure S20. FT-EXAFS fitting curves of Pt_1/NC. (a) k^2 weighted k-space. (b) k^2 weighted R-space.

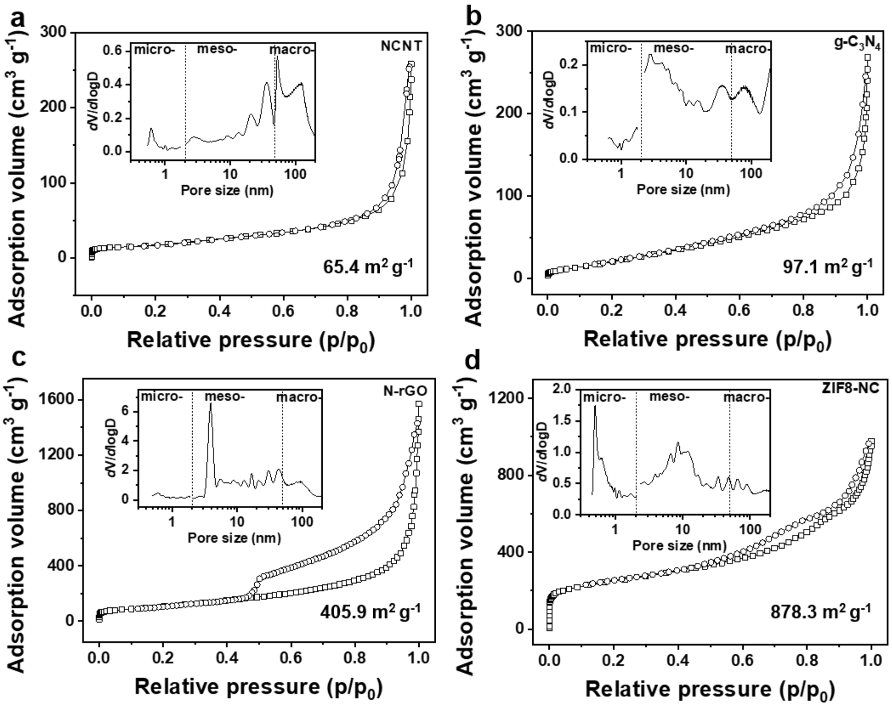

e

	NCNT	g-C_3N_4	N-rGO	ZIF8- NC	hNCNC
SSA (m^2 g^{-1})	65.4	97.1	405.9	878.3	611.0
N content (at.%)	2.3	52.2	7.1	7.2	9.2
Pt loading* (wt.%)	0.97 (Pt_1/NCNT)	0.76 (Pt_1/g-C_3N_4)	0.94 (Pt_1/N-rGO)	3.02 (Pt_1/ZIF8-NC)	3.0 (Pt_1/hNCNC)

*Theoretical Pt loading is 3 wt%.

Figure S21. N_2 adsorption-desorption isotherms and parameters of some nitrogen-doped carbon supports. (a-d) NCNT, g-C_3N_4, N-rGO and ZIF8-derived NC, respectively. Insets are the corresponding pore size distributions. (e) SSAs and N contents of the samples. Pt loadings in corresponding SACs measured by ICP-OES are listed for convenient comparison.

The microfluidic synthesis of noble metal SACs depends on the dual-scale cooperation between macroscopic fluid dynamics and microscopic support chemistry. Overall, the validity of this method relies on three key factors.

1) Precursor compatibility: This method relies on anionic noble metal chloride precursors that form stable complexes under acidic conditions. Electrostatic attraction between anions such as $[PtCl_6]^{2-}$ and $[PdCl_4]^{2-}$ and protonated pyridinic-N sites plays a key role in promoting rapid adsorption (Fig. 3e,f, Figs. S13, S14, Table S4). This method is not directly applicable to cationic or neutral metal complexes, which typically require high-temperature treatment [5,34].

2) Support requirements: Abundant micropores with matched sizes and N-doping sites (especially pyridinic N) are required to enable synergistic physical confinement and chemical anchoring. Supports without these features tend to yield low loadings or form nanoparticles. Here, the near-theoretical loading on ZIF8-NC is ascribed to its similar large specific surface area (SSA), pore size distribution and high N content to hNCNC, while the lower loadings on other supports are likely due to the factors such as lower SSA, pore size mismatch, or insufficient N content.

3) Process parameters: Precursor concentration, support concentration, flow rate, and temperature should be optimized for each metal/support system to ensure uniform adsorption and suppress aggregation.

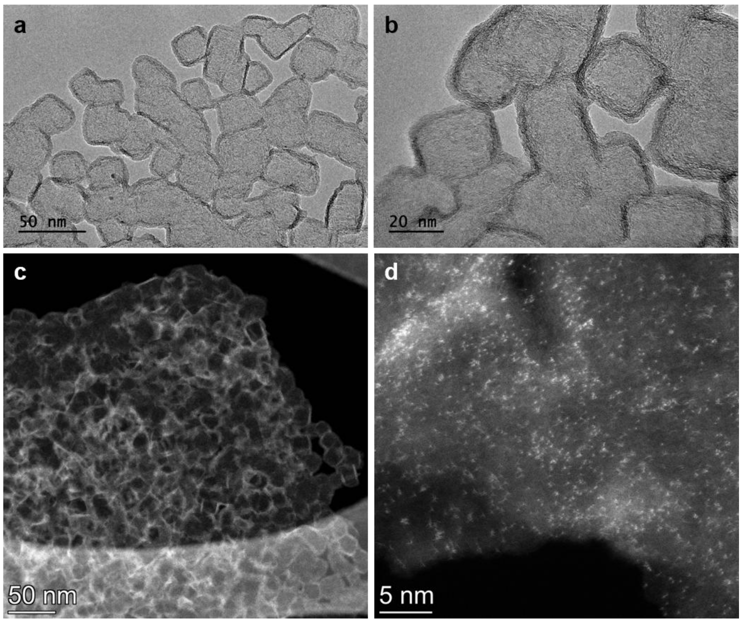

Figure S22. TEM (a,b) and HAADF-STEM (c,d) images of 10 wt%-Pt_1/hNCNC after 10000 CV cycles.

After 10000 CV cycles of ADT, Pt_1/hNCNC maintains Pt single atoms without observable morphological change.

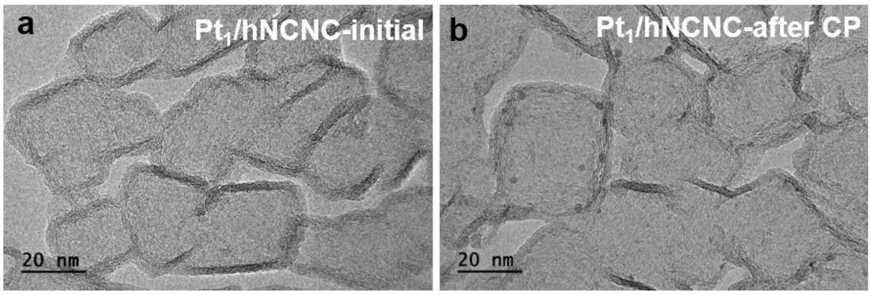

Figure S23. TEM images of Pt_1/hNCNC before (a) and after (b) 500 h chronopotentiometry test.

After 500 h test, slight aggregation was observed which should be the reason for slight voltage increase in Fig. 5i.

Note S1. Continuous-flow strategy offers favorable scalability, cost-effectiveness, and operational safety.

1) Scalability. The modular nature of microfluidic reactors allows facile scale-up via numbering-up parallelization rather than enlarging reactor size. Our current single module yields 30 g/day. Scaling to 30-40 parallel modules readily achieves kg-scale daily output without sacrificing product uniformity.

2) Manufacturing cost. The simple equipment (peristaltic pump, PTFE tubing) reduces capital investment. Moreover, this method significantly reduces labor costs via continuous operation and minimizes energy consumption due to efficient heat transfer. High Pt utilization (up to 10 wt% single-atom loading) and ultralow device loading (20 μg_{Pt} cm^{-2}) further lower overall material cost.

3) Operational safety. Synthesis proceeds under mild conditions (60°C, ambient pressure), avoiding the high temperatures or pressures often required in other methods. Product isolation requires only simple filtration and drying, eliminating complex or hazardous post-treatment steps. These features collectively ensure a high level of operational safety.

Table S1. Synthesis conditions of different samples.

Catalyst	Metal precursor	pH	Precursor concentration (mg mL^{-1})	Support concentration (mg mL^{-1})	Temperature (°C)	Flow rate (mL min^{-1})
3 wt%-Pt_1/hNCNC	H_2PtCl_6	1	0.0996	1.5	60	15
10 wt%-Pt_1/hNCNC	H_2PtCl_6	1	0.1385	0.625		5
Pd_1/hNCNC	$PdCl_2$	1	0.0213			
Au_1/hNCNC	$HAuCl_4$	0	0.0220			
Ir_1/hNCNC	H_2IrCl_6	1	0.0198			
Rh_1/hNCNC	$RhCl_3$	1	0.0259			
Ru_1/hNCNC	$RuCl_3$	1	0.0262			
HESAC	$Co(NO_3)_2$<br>$Ni(NO_3)_2$<br>$Cr(NO_3)_3$<br>$Mn(NO_3)_2$	0	0.50			
	H_2PtCl_6<br>$PdCl_2$<br>$HAuCl_4$<br>H_2IrCl_6<br>$RhCl_3$<br>$RuCl_3$	0	0.70			
Pt_1/NCNT	H_2PtCl_6	1	0.0415			
Pt_1/g-C_3N_4	H_2PtCl_6	1	0.0415			
Pt_1/N-rGO	H_2PtCl_6	1	0.0415			
Pt_1/ZIF8-NC	H_2PtCl_6	1	0.0415			

Table S2. EXAFS fitting parameters for various samples (S_0^2=0.89).

Sample	Shell	*CN* [a]	*R* (Å) [b]	σ^2×10^3 (Å^2) [c]	ΔE_0 (eV) [d]	*R* factor
Pt foil	Pt-Pt	12*	2.77±0.01	5.0±0.8	7.7±0.3	0.001
PtO_2	Pt-O	5.6±0.5	2.02±0.01	1.9±0.5	11.4±1.1	0.007
	Pt-Pt	7.0±1.0	3.10±0.01	3.1±0.5	10.6±1.1	
	Pt-Pt	3.5±1.5	3.84±0.03	31.3±7.5		
Pt_1/hNCNC	Pt-C/N	2.252	1.99±0.04	2.22±1.3	11.2±2.6	0.009
	Pt-Cl	2.189	2.30±0.03	13.4±2.2		

[a]*CN*: coordination numbers; [b]*R*: bond distance; [c]σ^2: Debye-Waller factors; [d]ΔE_0: inner potential correction. *R* factor: goodness of fit. S_0^2 was set to 0.89 according to the experimental EXAFS fit of Pt foil by fixing *CN* as the known crystallographic value. Error bounds (accuracies) that characterize the structural parameters obtained by EXAFS spectroscopy were estimated as *CN* ± 20%; *R* ± 1%; σ^2± 20%; ΔE_0 ± 20%. The k^2 weighting, *k*-range of 3.0~10 Å^{-1} and *R* range of 1.0~3.0 Å were used for the fitting. The model of Pt-N/C were used to calculate the simulated scattering paths. The four parameters, coordination number, bond length, Debye-Waller factor and E_0 shift (*CN*, *R*, σ^2, ΔE_0) was partial fixed.

Table S3. Calculation parameters for the FEM model.

Parameter	Description	Value
v_0	velocity	4.7× 10^{-2} m s^{-1}
D	bulk diffusion coefficient	1.4 × 10^{-9} m^2 s^{-1}
D_s	surface diffusion coefficient	1.0 × 10^{-16} m^2 s^{-1}
k_{ads}	adsorption rate constant	8.0 × 10^{-8} m^3 (s·mol)$^{-1}$
k_{des}	desorption rate constant	8.0 × 10^{-11} s^{-1}
Γ_s	saturation adsorption site density	1.0 × 10^4 mol m^{-2}

Notes: c_0 denotes the $[PtCl_6]^{2-}$ ion concentration at the left boundary of microelements. For the first microelement at the inlet of the spiral flow reactor, c_0 is set as 1.321 mol m^{-3} according to our experimental condition. v_0 is calculated by v_0 = Q/A, where Q = 20 × 10^{-6} m^3/s is the volumetric flow rate, A = πr^2 is the cross-sectional area of the channel with r = 3 mm. D is taken as a reference value for the bulk diffusion coefficient, consistent with the order of magnitude for various small inorganic anions in aqueous solution. D_s, k_{ads}, k_{des}, and Γ_s are estimated parameters chosen such that the outlet concentration is approximately zero when the inlet concentration is 1.0 mol m^{-3}.

Table S4. The adsorption energies [E_{ab} (eV)] of different anions on/inside five models.

	**Model-1**	**Model-2**	**Model-3**	**Model-4**	**Model-5**
$[PtCl_6]^{2-}$	-3.48	-4.47	-4.88	-5.25	-5.78
$[PdCl_4]^{2-}$	-2.97	-3.61	-4.00	-4.39	-5.01
$[AuCl_4]^{-}$	-2.88	-3.20	-3.42	-3.73	-4.33
$[IrCl_6]^{2-}$	-2.85	-3.80	-4.66	-4.87	-5.46
$[RhCl_6]^{3-}$	-3.78	-4.76	-5.16	-5.64	-6.34
$[RuCl_6]^{3-}$	-2.98	-3.83	-4.14	-4.59	-5.23

Table S5. Comparison of the PEMWE performance with reported Pt-based catalysts.

**Catalyst**	**Activity**	**Pt loading ($\mu g\ cm^{-2}$)**	**Membrane**	**Reference**
**Pt_1/hNCNC**	**1.66 V@1 A cm^{-2}**<br>**2.00 V@3 A cm^{-2}**	**20**	**Nafion 115**	**This work**
Pt_1/hNCNC	1.66 V@1 A cm^{-2}	25	Nafion 115	[7]
Pt-MoC_x@C	2.0 V@1 A cm^{-2}	53	Nafion 115	[36]
Pt/B–$WO_{2.9}$	1.78 V@1 A cm^{-2}	500	Nafion 115	[37]
I-PtZn@NPC	1.60 V@1 A cm^{-2}	600	Nafion 115	[38]
Alloyed Pt SA	1.82 V@1 A cm^{-2}	20	Nafion 117	[39]
Mo_2TiC_2-Pt_{NC}	1.65 V@1 A cm^{-2}	36	Nafion 117	[40]
Pt_{SA}, P/np-MoS_2	1.67 V@1 A cm^{-2}	-	Nafion 117	[41]
20 wt% Pt	1.5 V@ 1 A cm^{-2}	-	Nafion 117	[42]
20 wt% Pt/C	1.95 V@1 A cm^{-2}	650	Nafion 117	[43]
30 wt% Pt/C	1.80 V@2.6 A cm^{-2}	500	Nafion 115	[44]
40 wt% Pt/C	1.84 V@ 3 A cm^{-2}	1000	Nafion 115	[45]
47.7 wt% Pt/C	1.80 V@1.98 A cm^{-2}	400	Nafion 115	[46]
70 wt % Pt/C	2.0 V@2.2 A cm^{-2}	400	Nafion 115	[47]
Pt black	2 V@ 2.82 A cm^{-2}	3000	Nafion 115	[48]
Pt/C	1.83 V@2 A cm^{-2}	500	Nafion 115	[49]
40 wt% Pt	1.5 V@ 1 A cm^{-2}	200	Nafion 212	[46, 50]
46.5 wt% Pt/C	1.6 V@2 A cm^{-2}	500	Nafion 212	[51]
46.5 wt% Pt/C	1.6 V@ 0.96 A cm^{-2}	400	Nafion 212	[52]

References

1. Chen S, Bi J, Zhao Y *et al.* Nitrogen-Doped Carbon Nanocages as Efficient Metal-Free Electrocatalysts for Oxygen Reduction Reaction. *Adv Mater* 2012; **24**: 5593–5597.
2. Chen H, Yang Y, Hu Z *et al.* Synergism of C_5N Six-Membered Ring and Vapor−Liquid−Solid Growth of CN_x Nanotubes with Pyridine Precursor. *J Phys Chem B* 2006; **110**: 16422–16427.
3. Li JJ, Banis MN, Ren ZH *et al.* Unveiling the Nature of Pt Single-Atom Catalyst during Electrocatalytic Hydrogen Evolution and Oxygen Reduction Reactions. *Small* 2021; **17**: 2007245.
4. Mou Z, Chen X, Du Y *et al.* Forming Mechanism of Nitrogen Doped Graphene Prepared by Thermal Solid-State Reaction of Graphite Oxide and Urea. *Appl Surf Sci* 2011; **258**: 1704–1710.
5. Chen Y, Zhang J, Tian J *et al.* Hierarchical Ni/N/C Single-Site Catalyst Achieving Industrial-Level Current Density and Ultra-Wide Potential Plateau of High CO Faradic Efficiency for CO_2 Electroreduction. *Adv Funct Mater* 2023; **33**: 2214658.
6. Shi YX, Wu Y, Wang SQ *et al.* Soft Electrochemical Actuators with a Two-Dimensional Conductive Metal-Organic Framework Nanowire Array. *J Am Chem Soc* 2021; **143**: 4017–4023.
7. Tian J, Xia M, Cheng X *et al.* Understanding Pt Active Sites on Nitrogen-Doped Carbon Nanocages for Industrial Hydrogen Evolution with Ultralow Pt Usage. *J Am Chem Soc* 2024; **146**: 33640–33650.
8. Delley B. An All-Electron Numerical-Method for Solving the Local Density Functional for Polyatomic-Molecules. *J Chem Phys* 1990; **92**: 508–517.
9. Perdew JP, Burke K, Ernzerhof M. Generalized Gradient Approximation Made Simple. *Phys Rev Lett* 1996; **77**: 3865–3868.
10. Klamt A, Schuurmann G. Cosmo - A New Approach to Dielectric Screening in Solvents with Explicit Expressions for the Screening Energy and Its Gradient. *J Chem Soc, Perkin Trans 2* 1993: 799–805.
11. Mulliken RS. Electronic Population Analysis on Lcao-Mo Molecular Wave Functions. I. *J Chem Phys* 1955; **23**: 1833–1840.
12. Mulliken RS. Electronic Population Analysis on Lcao-Mo Molecular Wave Functions. II. Overlap Populations, Bond Orders, and Covalent Bond Energies. *J Chem Phys* 1955; **23**: 1841–1846.
13. Zhang Z, Chen Y, Zhou L *et al.* The Simplest Construction of Single-Site Catalysts by the Synergism of Micropore Trapping and Nitrogen Anchoring. *Nat Commun* 2019; **10**: 1657.
14. Kühne TD, Iannuzzi M, Del Ben M *et al.* CP2K: An electronic structure and molecular dynamics software package - Quickstep: Efficient and accurate electronic structure calculations. *J Chem Phys* 2020; **152**: 194103.
15. Hutter J, Iannuzzi M, Schiffmann F *et al.* CP2K: atomistic simulations of condensed matter systems. *Wires Comput Mol Sci* 2014; **4**: 15–25.
16. Grimme S, Antony J, Ehrlich S *et al.* A consistent and accurate ab initio parametrization of density functional dispersion correction (DFT-D) for the 94 elements H-Pu. *J Chem Phys* 2010; **132**: 154104.
17. Perdew JP, Burke K, Ernzerhof M. Generalized gradient approximation made simple. *Phys Rev Lett* 1997; **78**: 1396.
18. Hartwigsen C, Goedecker S, Hutter J. Relativistic separable dual-space Gaussian pseudopotentials from H to Rn. *Phys Rev B* 1998; **58**: 3641–3662.
19. Goedecker S, Teter M, Hutter J. Separable dual-space Gaussian pseudopotentials. *Phys Rev B* 1996; **54**: 1703–1710.
20. Nose S. A Molecular-Dynamics Method for Simulations in the Canonical Ensemble. *Mol Phys* 1984; **52**: 255–268.

21. Nose S. A Unified Formulation of the Constant Temperature Molecular-Dynamics Methods. *J Chem Phys* 1984; **81**: 511–519.
22. Thompson AP, Aktulga HM, Berger R *et al.* LAMMPS-A Flexible Simulation Tool for Particle-Based Materials Modeling at the Atomic, Meso, And Continuum Scales. *Comput Phys Commun* 2022; **271**: 108171.
23. Planck M. Ueber die Erregung von Electricität und Wärme in Electrolyten. *Ann Phys* 1890; **275**: 161–186.
24. He YF, Yazhgur P, Salonen A *et al.* Adsorption-desorption kinetics of surfactants at liquid surfaces. *Adv Colloid Interfac* 2015; **222**: 377–384.
25. Wu Q, Yang LJ, Wang XZ *et al.* Carbon-Based Nanocages: A New Platform for Advanced Energy Storage and Conversion. *Adv Mater* 2020; **32**: 1904177.
26. Wu Y, Yang Y, Zhao X *et al.* A Novel Hierarchical Porous 3D Structured Vanadium Nitride/Carbon Membranes for High-performance Supercapacitor Negative Electrodes. *Nano-Micro Lett* 2018; **10**: 63.
27. Chen S, Luo T, Li X *et al.* Identification of the Highly Active Co-N_4 Coordination Motif for Selective Oxygen Reduction to Hydrogen Peroxide. *J Am Chem Soc* 2022; **144**: 14505–14516.
28. Chen Y, Ding R, Li J *et al.* Highly active atomically dispersed platinum-based electrocatalyst for hydrogen evolution reaction achieved by defect anchoring strategy. *Appl Catal, B* 2022; **301**: 120830.
29. Jiang D, Yuan HF, Liu Z *et al.* Defect-Anchored Single-Atom-Layer Pt Clusters on TiO_{2-x}/Ti for Efficient Hydrogen Evolution via Photothermal Reforming Plastics. *Appl Catal, B* 2023; **339**: 123081.
30. Hui L, Zhang XT, Xue YR *et al.* Highly Dispersed Platinum Chlorine Atoms Anchored on Gold Quantum Dots for a Highly Efficient Electrocatalyst. *J Am Chem Soc* 2022; **144**: 1921–1928.
31. Wang N, Zhao XH, Zhang R *et al.* Highly Selective Oxygen Reduction to Hydrogen Peroxide on a Carbon-Supported Single-Atom Pd Electrocatalyst. *ACS Catal* 2022; **12**: 4156–4164.
32. Liu H, Zou HY, Wang D *et al.* Second Sphere Effects Promote Formic Acid Dehydrogenation by a Single-Atom Gold Catalyst Supported on Amino-Substituted Graphdiyne. *Angew Chem Int Edit* 2023; **62**: e202216739.
33. Zhu L, Ma CL, Li DN *et al.* Designing of Hexagonal Nanosheets with Edge-Sharing [IrO_6] Octahedral Crystals for Efficient and Stable Acidic Water Splitting. *Adv Funct Mater* 2024; **34**: 2313375.
34. Xiong Y, Dong JC, Huang ZQ *et al.* Single-atom Rh/N-doped carbon electrocatalyst for formic acid oxidation. *Nat Nanotechnol* 2020; **15**: 390–397.
35. Sun ZX, Zhou CK, Chen ZJ *et al.* Decoupling Activity-Selectivity Trade-off in Photothermal Catalytic CO_2 Hydrogenation: A Hydrogen Spillover-Assisted Dual-Site Synergy Mechanism. *Angew Chem Int Edit* 2025: e202508090.
36. Chen LL, Huang YC, Ding YP *et al.* Interfacial engineering of atomic platinum-doped molybdenum carbide quantum dots for high-rate and stable hydrogen evolution reaction in proton exchange membrane water electrolysis. *Nano Res* 2023; **16**: 12186–12195.
37. Qu MR, Cheng YX, Feng SH *et al.* Ordered interfacial domain expansion catalysis enhances hydrogen evolution for proton exchange membrane electrolysis. *Energ Environ Sci* 2025; **18**: 5985–5997.
38. Wang ML, Shi ZJ, Shi WW *et al.* Ultrafast H-Spillover in Intermetallic PtZn Induced by the Local Disorder for Excellent Electrocatalytic Hydrogen Evolution Performance. *Adv Mater* 2025; **37**: 2409575.
39. Gao H, Jiang Y, Chen R *et al.* Alloyed Pt Single-Atom Catalysts for Durable PEM Water Electrolyzer. *Adv Funct Mater* 2023; **33**: 2214795.
40. Zeng HL, Chen Z, Jiang Q *et al.* Sustainable and cost-efficient hydrogen production using platinum

clusters at minimal loading. *Nat Commun* 2025; **16**: 4314.
41. Peng J, Wang Z, Jiang K *et al.* Phosphorus dopants triggered single-atom platinum catalysis for efficient hydrogen evolution in proton exchange membrane electrolyzers. *J Mater Chem A* 2024; **12**: 17395–17403.
42. Hao S, Liu M, Pan J *et al.* Dopants fixation of Ruthenium for boosting acidic oxygen evolution stability and activity. *Nat Commun* 2020; **11**: 5368.
43. Wu Z-Y, Chen F-Y, Li B *et al.* Non-iridium-based electrocatalyst for durable acidic oxygen evolution reaction in proton exchange membrane water electrolysis. *Nat Mater* 2022; **22**: 100–108.
44. Siracusano S, Van Dijk N, Payne-Johnson E *et al.* Nanosized IrO_x and $IrRuO_x$ electrocatalysts for the O_2 evolution reaction in PEM water electrolysers. *Appl Catal, B* 2015; **164**: 488–495.
45. Shi Z, Li J, Jiang J *et al.* Enhanced Acidic Water Oxidation by Dynamic Migration of Oxygen Species at the Ir/Nb_2O_{5-x} Catalyst/Support Interfaces. *Angew Chem Int Edit* 2022; **61**: e202212341.
46. Islam J, Kim S-K, Thien PT *et al.* Enhancing the activity and durability of iridium electrocatalyst supported on boron carbide by tuning the chemical state of iridium for oxygen evolution reaction. *J Power Sources* 2021; **512**: 230506.
47. Jiang G, Yu H, Li Y *et al.* Low-Loading and Highly Stable Membrane Electrode Based on an $Ir@WO_xNR$ Ordered Array for PEM Water Electrolysis. *ACS Appl Mater Interfaces* 2021; **13**: 15073–15082.
48. Doan TL, Lee HE, Kim M *et al.* Influence of IrO_2/TiO_2 coated titanium porous transport layer on the performance of PEM water electrolysis. *J Power Sources* 2022; **533**: 231370.
49. Hegge F, Lombeck F, Cruz Ortiz E *et al.* Efficient and Stable Low Iridium Loaded Anodes for PEM Water Electrolysis Made Possible by Nanofiber Interlayers. *ACS Appl Energy Mater* 2020; **3**: 8276–8284.
50. Wen Y, Chen P, Wang L *et al.* Stabilizing Highly Active Ru Sites by Suppressing Lattice Oxygen Participation in Acidic Water Oxidation. *J Am Chem Soc* 2021; **143**: 6482–6490.
51. Pham CV, Bühler M, Knöppel J *et al.* IrO_2 coated TiO_2 core-shell microparticles advance performance of low loading proton exchange membrane water electrolyzers. *Appl Catal, B* 2020; **269**: 118762.
52. Choe S, Lee B-S, Cho MK *et al.* Electrodeposited IrO_2/Ti electrodes as durable and cost-effective anodes in high-temperature polymer-membrane-electrolyte water electrolyzers. *Appl Catal, B* 2018; **226**: 289–294.
